# Nuclear receptors connect progenitor transcription factors to cell cycle control

**DOI:** 10.1038/s41598-017-04936-7

**Published:** 2017-07-07

**Authors:** Marta Neto, Marina Naval-Sánchez, Delphine Potier, Paulo S. Pereira, Dirk Geerts, Stein Aerts, Fernando Casares

**Affiliations:** 1 CABD, Andalusian Centre for Developmental Biology, CSIC-UPO-JA, 41013 Seville, Spain; 2 School of Medicine, University of Leuven, box 602 3000, Leuven, Belgium; 3 i3S - Instituto de Investigação e Inovação em Saúde, Universidade do Porto, Rua Alfredo Allen, 208, 4200-135 Porto, Portugal; 4 IBMC - Instituto de Biologia Molecular e Celular, Universidade do Porto, Rua Alfredo Allen, 208, 4200-135 Porto, Portugal; 5 Department of Medical Biology L2-109, Academic Medical Center, University of Amsterdam, Amsterdam, The Netherlands

## Abstract

The specification and growth of organs is controlled simultaneously by networks of transcription factors. While the connection between these transcription factors with fate determinants is increasingly clear, how they establish the link with the cell cycle is far less understood. Here we investigate this link in the developing *Drosophila* eye, where two transcription factors, the MEIS1 homologue *hth* and the Zn-finger *tsh*, synergize to stimulate the proliferation of naïve eye progenitors. Experiments combining transcriptomics, open-chromatin profiling, motif analysis and functional assays indicate that these progenitor transcription factors exert a global regulation of the proliferation program. Rather than directly regulating cell cycle genes, they control proliferation through an intermediary layer of nuclear receptors of the ecdysone/estrogen-signaling pathway. This regulatory subnetwork between *hth*, *tsh* and nuclear receptors might be conserved from *Drosophila* to mammals, as we find a significant co-overexpression of their human homologues in specific cancer types.

## Introduction

The programs for organ development are encoded in organ specification networks. In these networks, transcription factors (TFs) tightly control the specification of progenitor cells and their proliferation to ensure that the right types and amounts of cells are produced. Several organ-specification networks have been described in detail in the past years in vertebrates and invertebrates^1–6^. However, how transcription factors act upon the cell cycle machinery to regulate progenitor cell proliferation is still poorly understood. To investigate this issue we have resorted to the developing *Drosophila* eye, for which a detailed transcriptional network is available^6–8^.

In the fly eye primordium, eye progenitors are specified by the co-expression of a set of transcription factors: the two *Drosophila* Pax6 genes *eyeless* (*ey*) and *twin of eyeless* (*toy*), the TALE-class homeodomain *homothorax* (*hth*) and the Zn-finger encoding gene *teashirt* (*tsh*)^6, 9, 10^. This gene expression combination is transient: the undifferentiated, proliferative state of progenitors is maintained as long as they express *hth*. Accordingly, the forced maintenance of *hth* blocks retina differentiation. In its progenitor role, *hth* is known to interact with *tsh* ^10^. One important aspect of this interaction is that it is synergistic. Maintenance of *hth* stalls differentiation, while maintaining *tsh* only causes a mild retinal differentiation impairment (see below). However, maintaining the expression of both TFs (“hth + tsh”) results in large tumor-like overgrowths formed by progenitor-like cells^10, 11^. (see Results below). This suggests that hth + tsh together control the cell cycle machinery, directly or indirectly.

Interestingly, the vertebrate *hth* homologues, the MEIS gene family, not only are progenitor transcription factors that play essential roles during development^12–17^, but their elevated expression has been associated to a number of tumor types in mice and humans^18–28^.

The synergistic growth-inducing ability of Hth and Tsh has been attributed, at least in part, to their direct protein interaction with Yki^11^, the nuclear effector and transcriptional co-activator of the Salvador-Warts-Hippo tumor suppressor pathway^29^. Only one direct transcriptional target of the Hth:Tsh:Yki complex has been functionally validated to date, though, the miRNA-encoding gene *bantam* (*ban*)^11^. Even though *ban* is expected to have a rather pleiotropic effect^30, 31^, on its own it does not account for the large overgrowths induced by Hth:Tsh:Yki. Therefore, a global picture of the transcriptional changes specifically induced by *hth* and *tsh* and how these are connected to tissue growth is still lacking.

Here we have analyzed the global impact that hth + tsh have on the developing eye to establish links between these transcription factors and target genes, using genome-wide gene expression and open chromatin profiling, together with computational methods. The resulting picture of this epigenomic analysis is one in which the up-regulation of large numbers of cell cycle genes may be due to the primary regulation of a few potentially direct targets, that include a subset of components of the ecdysone/estrogen pathway. These nuclear receptors would then amplify the hth + tsh response by directly affecting cell cycle genes, as these genes show a signature of nuclear receptor binding. We tested this prediction functionally and showed that indeed *EcR*, the ecdysone receptor, and the nuclear receptors *Hr46*/*DHR3* and *ftz*-*f1* were required for hth + tsh-driven tumor-like overgrowth. Therefore, growth control by progenitor TFs Hth and Tsh would be funneled through nuclear receptors, acting as intermediaries. The connection between *hth*, *tsh* and nuclear receptors might extend beyond *Drosophila*, as we found a significant co-overexpression of the human homologues of *hth*, *tsh* and *ftz*-*f1*, the MEIS1, TSHZ and NRA52 genes, in specific cancer cell lines and tumors.

## Results

### Perturbations of progenitor transcription factors result in tissue overgrowth

**Figure 1:**
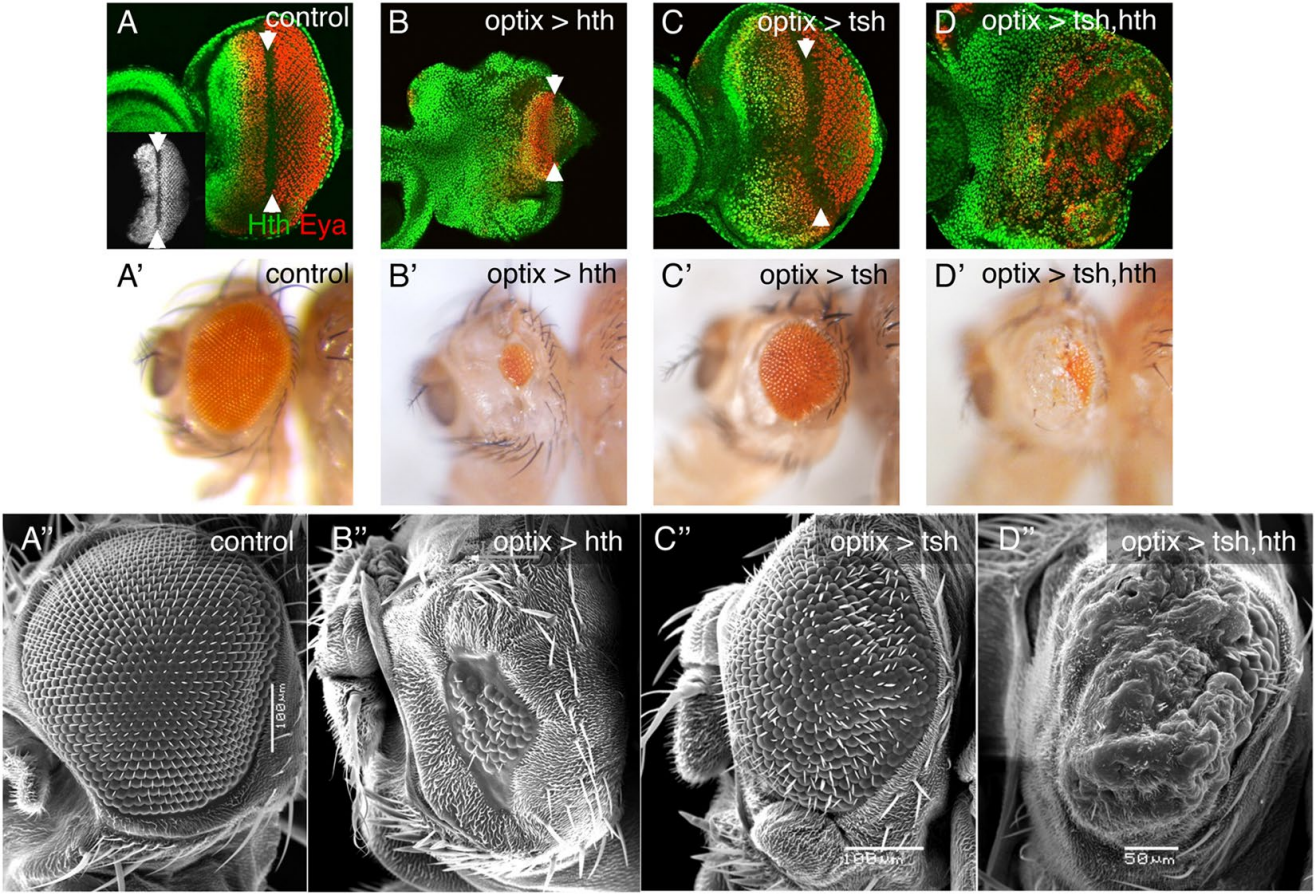
Forced maintenance of *hth and tsh* expression results in overgrowth and differentiation arrest. Late third instar (L3) eye discs (**A**–**D**) and adult heads (**A’**–**D’** and **A”**–**D”**) from control (*optix* > *GFP*) and hth- (*optix* > *hth*), tsh- (*optix* > *tsh*) or hth + tsh- (*optix* > *hth* + *tsh*) expressing animals. The GFP expression driven by *optix* > (*optix2*/*3*-*GAL4*; *UAS*-*GFP*) is shown in the inset in (**A**). Discs are stained with anti-Eya and anti-Hth. Anterior is left, dorsal is up (this orientation will be maintained throughout). (**A’**–**D’**) Lateral views of adult heads of the same genotypes as above. (**A”**–**D”**) SEM images of lateral views of adult heads of the corresponding genotypes. Overexpression of *hth* (*optix* > hth) results in a reduced eye disc area and smaller adult eye (**B**,**B”**). *tsh*-overexpressing flies (*optix* > tsh) show almost normal discs and retinal morphology (**C**–**C”**). However, overexpression of hth and tsh (*optix* > *hth* + *tsh*), results in overgrown eye discs showing abnormal folds. Adult heads develop a small retinal patch and an overgrowth of indistinct cuticle (**D**,**D”**).

During development, the expression of eye progenitor transcription factors is transient to allow cell cycle stop and differentiation. However, the forced maintenance in the eye primordium of two of these transcription factors simultaneously, *hth* and *tsh*, cause the tumor-like overgrowth of progenitor-like cells^10^. To analyze how the combined expression of these two factors drives proliferation, we expressed *hth* and *tsh*, either alone or in combination, using the eye-specific GAL4 driver *optix2*/*3*-*GAL4* (or “optix>”). *optix*> is specifically active in undifferentiated cells of the eye disc (Fig. 1A and Suppl. Figure 1 and ref. 32). Therefore, the effects of gene manipulations driven by *optix>* reflect effects on the undifferentiated population. While when *hth* or *tsh* were expressed alone no overproliferation was produced (*optix* > *GFP*:*hth* and *optix* > *tsh*, respectively; Fig. 1A–C), coexpression of both TFs in *optix* > *GFP*:*hth* + *tsh* (“hth + tsh”) produced disc overgrowths. The overgrown tissue does not differentiate into retina. This resulted, in the adult, in heads with a very small eyes surrounded by overgrown indistinct cuticle (Fig. 1A’,A”–D’,D”).

### Cell cycle genes and nuclear receptors are altered downstream of Hth + Tsh

**Figure 2:**
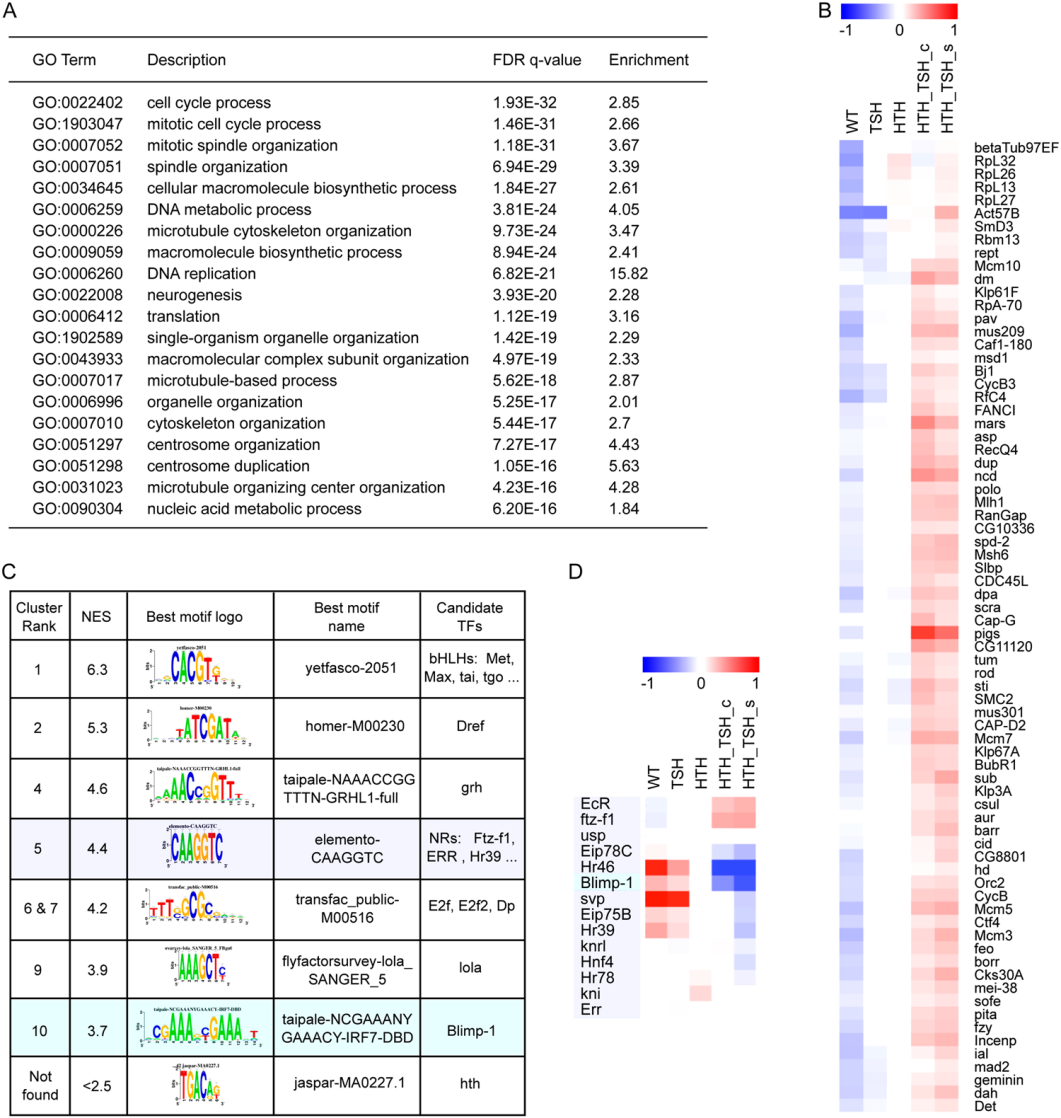
Transcriptomic profile of of hth + tsh cells. (**A**) Gene Ontology enrichment of genes up-regulated in hth + tsh compared to control eye discs (EA). Analysis performed by GOrilla^97^ on a ranked list of genes sorted by (signed) −log10(p-value). The sign indicates that up-regulated genes are on top (logFC > 0) and down-regulated genes (logFC < 0) are on the bottom of the list. (**B**) Heatmap with row-normalized expression values of the most significantly up-regulated cell-cycle related genes. (**C**) Motif enrichment on the up-regulated genes (770 genes, selected as the “leading edge” of the GOrilla analysis for cell cycle enrichment). Enrichment analysis is performed by i-cisTarget^33^ and enriched motifs are clustered within i-cisTarget using STAMP^103^. NES = Normalized Enrichment Score (>2.5 is significant). The Hth motif was not found enriched. (**D**) Heat map of expression profiles of motif related Nuclear Receptor genes and Blimp-1, showing strongest up-regulation of EcR and ftz-f1, and strongest down-regulation of Hr46 and Blimp-1.

To obtain a global view of the impact that hth + tsh co-expression had on gene expression, we generated the transcriptional profiles of late third larval stage (L3) eye discs from control (*optix* > *GFP*) as well as *hth*-expressing (*optix* > *GFP*:*hth*), *tsh*-expressing (*optix* > *tsh*), and *hth* + *tsh*-expressing (*optix* > *GFP*:*hth* + *tsh*) larvae using RNA-seq (see Materials and Methods). Principal component analysis of the RNA-seq data (Suppl. Fig. 2A) showed that *optix* > *tsh* clustered closest to the control, in agreement with its weak phenotype. *optix* > *GFP*:*hth* and the two *optix* > *GFP*:*hth* + *tsh* replicates were clearly distinguished. Next, when differential gene expression (DE) between the *optix* > *GFP*:*hth* + *tsh* and control samples was analyzed, the majority of DE-genes were down-regulated (Suppl. Fig. 2B). GO-enrichment analysis of the 503 significantly down-regulated genes (p.adj < 0.05 and log fold change (FC) < −1) identified “generation of neurons” and “compound eye photoreceptor cell differentiation” as enriched terms (Suppl. Fig. 2C), in agreement with the vestigial retina that develops in *optix* > *GFP*:*hth* + *tsh* adults. On the other hand, among the functions associated to the upregulated genes were those related to “cell cycle” and “DNA replication” (Suppl. Fig. 2D), which agree with the over-proliferative phenotype observed in *optix* > *GFP*:*hth* + *tsh* eye discs. This enrichment is found in a set of 103 significantly up-regulated genes (DE-seq, p.adj < 0.05 and logFC > 1), but is even stronger if the entire gene ranking is considered, whereby in the top 770 genes 74 cell cycle genes are recovered (p.adj 10^−32^, see Fig. 2A). From the heatmap shown in Fig. 2B it can be observed that the cell cycle-related genes are up-regulated as a consequence of the synergistic action of *hth* and *tsh*, since no up-regulation is observed when either *hth* or *tsh* are over-expressed alone. Among these genes we found key cell cycle regulators, such as *polo* kinase, *dp53* and *Rbf* and *Rbf2* (Fig. 2B). A list of DE-genes can be found in Suppl. Table 1.

In order to further analyse the effects of hth + tsh on cell cycle regulation, we generated *hth* + *tsh*-expressing clones and examined the expression of different cell cycle markers: the G1/S-phase *cyclin*-*E*, the G2/mitotic *cyclin*-*A* and the cyclin-dependent kinase inhibitor *dacapo* (*dap*). In agreement with their effect stimulating proliferation, hth + tsh coexpression induces *cyclin*-*A* and *cyclin*-*E* expression and represses *dap* expression in cell clones (Suppl. Fig. 3).

To identify transcription factors (TFs) that may control directly these DE genes we looked for TF binding site motif enrichment in the vicinity of the differentially expressed genes using i-*cis*Target^33, 34^ (See Materials and Methods). Down-regulated and up-regulated genes showed different motif enrichment: potential binding sites for E-box (top-enriched motif with a NES score of 4.73) and Glass (Gl) (motif also enriched with a NES score of 3.17) were found associated to down-regulated genes (data not shown). E-box-binding bHLH proteins Hairy, Daughterless, Emc and E(spl)-family members are known to participate in the specification of retinal precursors, regulating, among other genes, *atonal*, another bHLH transcription factor required for the specification of the R8 founder photoreceptor precursor^35–39^; *gl* encodes a five Zn-finger transcription factor required for the development of all photoreceptors^40^. These results were expected, since hth + tsh cause a blockade of the retinal developmental program.

On the other hand, up-regulated genes showed enrichment in potential binding sites for a bHLH TF (possibly Taiman), and for the general transcriptional co-factors Dref^41^ and Grainyhead^42^. Interestingly, motifs for E2F and nuclear hormone receptors are also strongly enriched, including EcR (Ecdysone receptor), ERR (estrogen-related receptor), ftz-f1, Hr46/DHR3 or Hr39 (Fig. 2C). E2F is necessary for normal proliferation and DNA synthesis^43–45^ and the enrichment in E2F potential target genes might reflect the vigorous proliferation of hth + tsh cells. The enrichment of binding sites for nuclear hormone receptors of the EcR pathway in up-regulated genes was unexpected, and suggests that a critical subset of the up-regulated genes could be under the direct control of nuclear hormone receptors.

**Figure 3:**
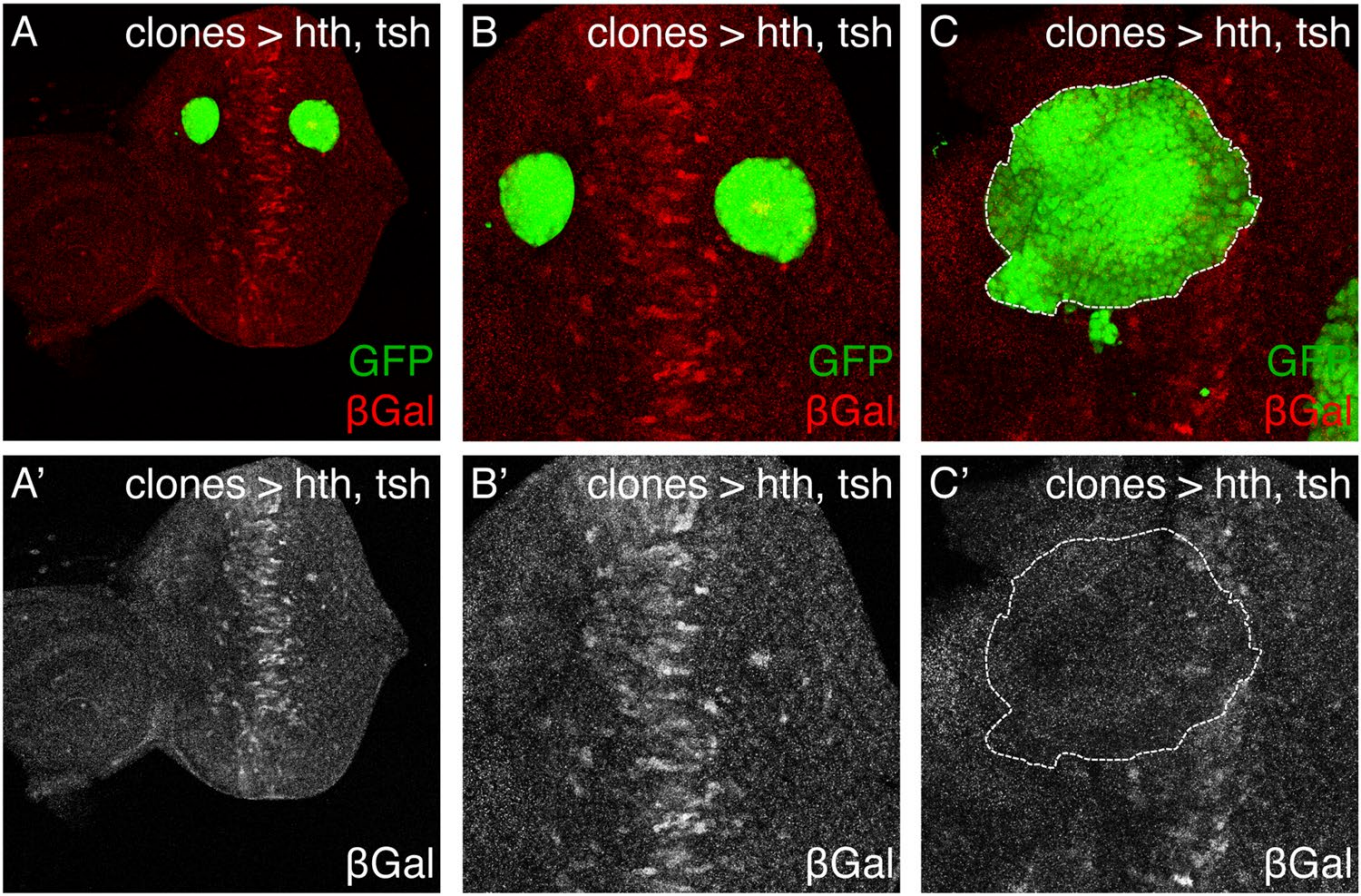
Co-expression of hth + tsh downregulate EcR signaling. hth + tsh-expressing clones (marked with GFP) induced in an Ecdysone Response Element-*lacZ* (EcRE-Z) background analyzed in L3 eye discs. *lacZ* expression is monitored with an anti-β galactosidase antibody (β gal). EcRE-Z is expressed straddling the morphogenetic furrow (dashed line) exclusively (**A**,**A’**). hth + tsh-clones overlapping the EcRE-Z domain repress its expression (**B**), while clones elsewhere do not (**A**,**A’**). (**A**) is a lower magnification view of the disc shown in (**A’**) where the whole pattern of EcRE-Z can be seen. The EcRE-Z sinla is shown separately in the lower panels.

The finding of EcR/nuclear receptor-related motifs prompted us to investigate the expression profile of the members of the EcR signaling cascade differentially expressed specifically in hth + tsh cells (Fig. 2D). These included the nuclear receptors EcR and ftz-f1 (up-regulated) and the nuclear receptor Hr46/DHR3 and the transcriptional repressor Blimp-1 (down-regulated), this latter also a regulator of the ecdysone pathway^46^. We noted that this pattern of nuclear receptor gene expression, characterized by high *EcR*/*ftz*-*f1* and low *Hr46*/*Blimp*-*1* is typical of a low/moderate ecdysone signaling^46–49^. Indeed, activity of the ecdysone pathway in L3 eye discs, monitored using an Ecdysone Response Element-lacZ (EcRE)^50^, can be observed straddling the morphogenetic furrow (MF), but not in more anterior regions, where the hth + tsh progenitor cells reside^51^. To test whether hth + tsh could reduce ecdysone signaling, we generated hth + tsh-expressing clones in an EcRE-Z background. Clones that span the MF show reduced EcRE-Z activity, while clones located elsewhere do not modify this reporter’s activity (Fig. 3A–C). Therefore, indeed co-expression of hth + tsh downregulates the response of cells to ecdysone signaling, and in a cell-autonomous manner.

Interestingly, we did not find an enrichment of Hth binding site (BS) motifs among the collection of hth + tsh DE genes (TGACA; http://pgfe.umassmed.edu/ffs/; note that a Tsh binding motif has not yet been described). The failure in finding Hth BSs could be explained by either one of two possibilities. First, Hth might bind to regulatory regions of many DE genes, but using a non-canonical BS. We find this unlikely, because all available experimental evidence (bacterial-1-hybrid in *Drosophila*, ChIP-seq in mouse, SELEX, protein binding microarrays, and manual curation) has retrieved the same binding motif for Hth/MEIS in invertebrates and vertebrates: the monomeric motif TGTCA or the palindromic dimer motif TGACA_NN_TGTCA^52^. Neither of these two motifs was found enriched in the up- or down-regulated DE gene set. We further noted that the DE genes regulated by hth + tsh were not enriched in Hth-binding sites previously identified using ChIP-seq^53, 54^. Alternatively, Hth might bind using its canonical BSs, but only on a relatively small subset of DE genes (primary targets), which then would amplify Hth regulation through secondary (indirect) targets. In such a situation, motif enrichment searches would not detect the Hth motif as significantly enriched. With this idea in mind, we looked for candidate direct targets by analyzing activity changes in associated regulatory regions.

### Open chromatin profiling confirms Nuclear Receptors as candidate regulators

**Figure 4:**
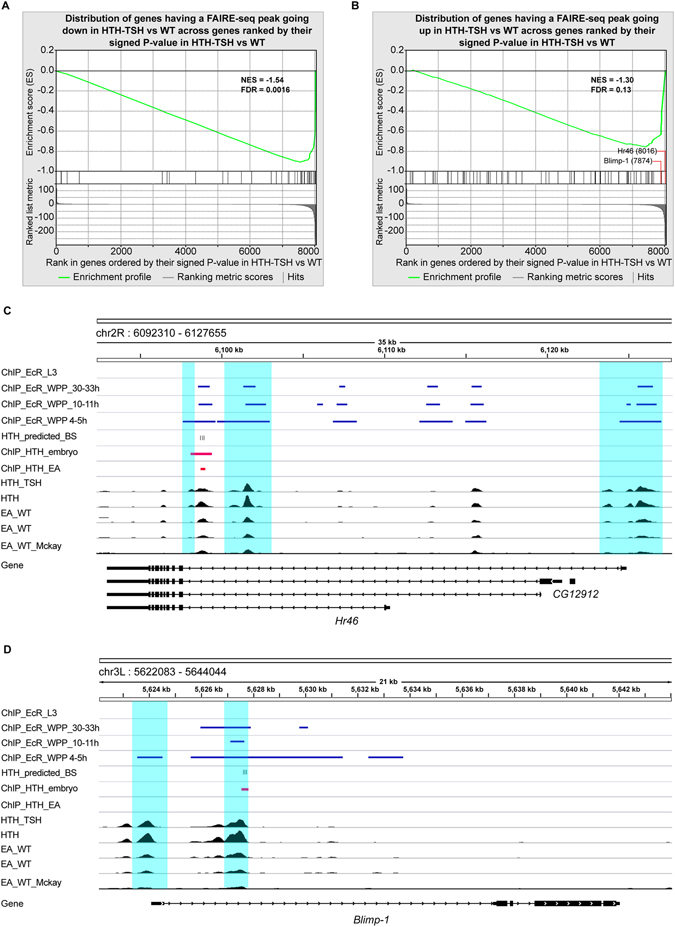
FAIRE-seq open chromatin profiling of hth + tsh cells. (**A**) Gene Set Enrichment Analysis (GSEA, ref. 100) compares gene expression changes with open chromatin changes. In the x-axis are all genes, ranked by the significance p-value of differential expression of control versus hth + tsh samples, with genes down-regulated in hth + tsh on the left, and genes up-regulated on the right. The tested gene sets (shown as black vertical lines) are genes with nearby (in 5 kb upstream and intronic space) FAIRE-seq peaks showing significant *decreased accessibility*. The correlation between both is highly significant (FDR < 0.001). (**B**) Similar plot, comparing changes in gene expression with genes showing nearby FAIRE-peaks with *increased accessibility*. In this case, the correlation is not significant, but the most down-regulated nuclear receptors Hr46 and Blimp-1 (indicated) are among the few genes showing peaks with increased accessibility. (**C**,**D**) Genomic view of Hr46 (**C**) and Blimp-1 (**D**) showing FAIRE-seq open chromatin profilling data for *optix* > *hth* + *tsh* (“HTH_TSH”), *optix* > *hth* (“HTH”) and control eye-antennal discs (EA) (EA_WT: black wiggle plot tracks); Hth ChIP-seq target regions in embryo and EA disc are shown with a red line; HTH-TSH versus WT differentially open chromatin peaks are highlighted with a cyan background; and prediction of binding sites within Hth ChIP peaks are shown as black ticks marked as “HTH_predicted_BS” (Cluster-Buster)^104^ motif score >6 using FlyFactorSurvey PWMs). In addition, ModENCODE EcR ChIP data are shown with a blue line, for L3 (modEncode_2640), WPP 4–5 h (modEncode_3398), WPP 10–11 h (modEncode_2641), WPP 30–33 h (modEncode_2642).

Accessible chromatin regions are associated to active promoters and *cis*-regulatory elements (CREs). Therefore, we reasoned that changes in the activity of distal CREs overlapping Hth binding sites (from Hth-ChIP data), and located near DE genes would point to hth + tsh direct targets. To this end, we carried out open chromatin profiling using FAIRE-seq^55–57^. Specifically, we compared the FAIRE-seq eye disc profiles of two control strains (see Materials and Methods) and *optix* > *GFP*:*hth*, *optix* > *tsh* and *optix* > *GFP*:*hth* + *tsh* (Suppl. Table 2 and Suppl. Fig. 4). We identified relatively few CREs with significantly altered chromatin accessibility. This finding was rather unexpected. First, the severe overgrowth phenotype and the large amount of differentially expressed genes suggested otherwise. Second, in another *Drosophila* model of eye overgrowth/cancer (induced by simultaneous expression of oncogenic *ras* and loss of *scribble*) dramatic chromatin changes have been described^57^. Specifically, with a particular set of stringent parameters, we identified only 86 CREs showing significantly increased accessibility when hth + tsh were co-expressed (log2(FC) > 1 and (p-adj < 0.05)), and 87 with significantly decreased accessibility (log2(FC) < 1 (p-adj < 0.05)). The regions with decreased accessibility are significantly associated with down-regulated genes (Fig. 4A), mostly related to the loss of the differentiation program in the eye disc. On the other hand, only a handful of regions with increased accessibility are associated with down-regulated genes (Fig. 4B), of which *Hr46* and *Blimp*-*1* are the most prominent examples (Fig. 4B). We did not find a significant association between peaks with increased accessibility and up-regulated genes (Fig. 4B). We next used i-*cis*Target to identify TF motifs within the FAIRE peaks showing increased accessibility, and again found Nuclear Receptor motifs (in this case, among NRs the EcR motif is the strongest, see Suppl. Fig. 5), but did not identify the Hth motif as enriched in the set. However, the few CREs with increased accessibility located near the down-regulated genes *Hr46* and *Blimp*-*1*, show overlapping or nearby ChIP-peaks for Hth (data from embryos^58^ and eye discs^59^ suggesting that hth + tsh may be directly repressing these nuclear receptor genes (Fig. 4C,D). Interestingly, these peaks also overlap EcR binding sites (Fig. 4C,D).

So far these data indicate that hth + tsh progenitor-like cells drive a specific pattern of nuclear receptor expression, characteristic of low/moderate ecdysone signaling, and that this expression pattern could be playing a direct role in the hth + tsh-induced overgrowths by in turn controlling a large set of cell cycle genes. If this hypothesis were true, some of these nuclear receptors should be required for the hth + tsh-driven tissue overgrowth. In addition, their expression should be regulated by hth + tsh *in vivo*. We next tested these two assumptions in turn.

### Functional analysis indicates that regulation of nuclear receptors EcR, ftz-f1 and Hr46/DHR3 controls hth + tsh-driven overgrowth

**Figure 5:**
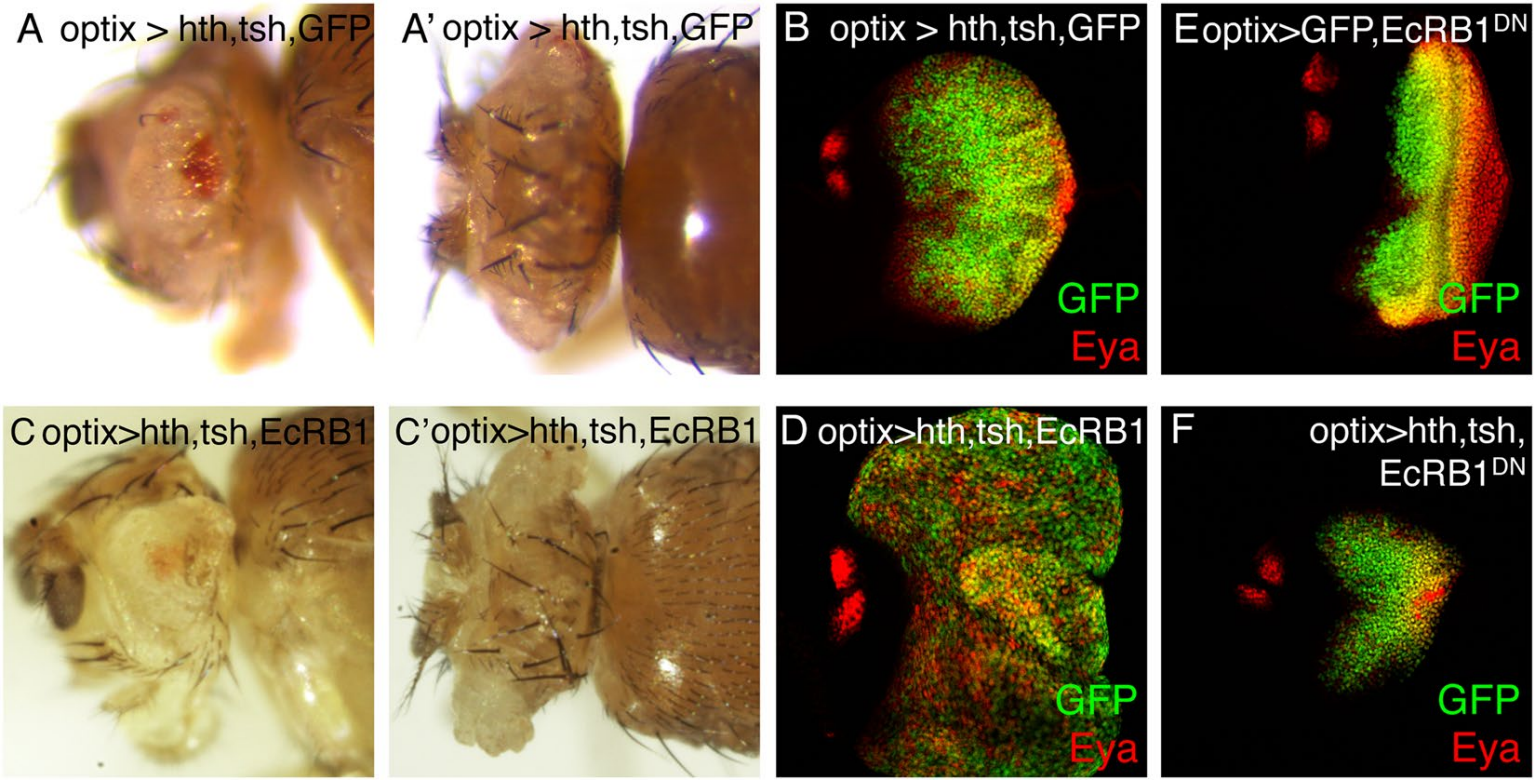
EcR functionally interacts with hth + tsh in inducing tissue overgrowth. Adult heads (**A**,**C** lateral and **A’**,**C’** dorsal views) and eye discs (**B**,**D**) of *optix* > *GFP*:*hth* + *tsh* + *GFP* (**A**,**B**) and *optix* > *GFP*:*hth* + *tsh* + *EcRB1* (**C**,**D**) (note that both genotypes harbor equal number of UAS-transgenes). Co-overexpression of EcRB1 enhances the overgrowth of lateral head cuticle and eye disc tissue. Comparison between eye discs overexpressing a dominant-negative form of the EcRB1 (**E**: *optix* > *GFP* + *EcRB1*-*DN*) and the co-overexpression of EcRB1-DN with hth + tsh (**F**: *optix* > *GFP*:*hth* + *tsh* + *EcRB1*-*DN*). Expression of EcRB1 causes a mild reduction in eye disc size (**E**). Coexpression of EcRB1-DN suppresses the overgrowth produced by hth + tsh (compare **F** with **B**). Discs are stained with anti-GFP (green) and anti-Eya (red) antibodies.

**Figure 6:**
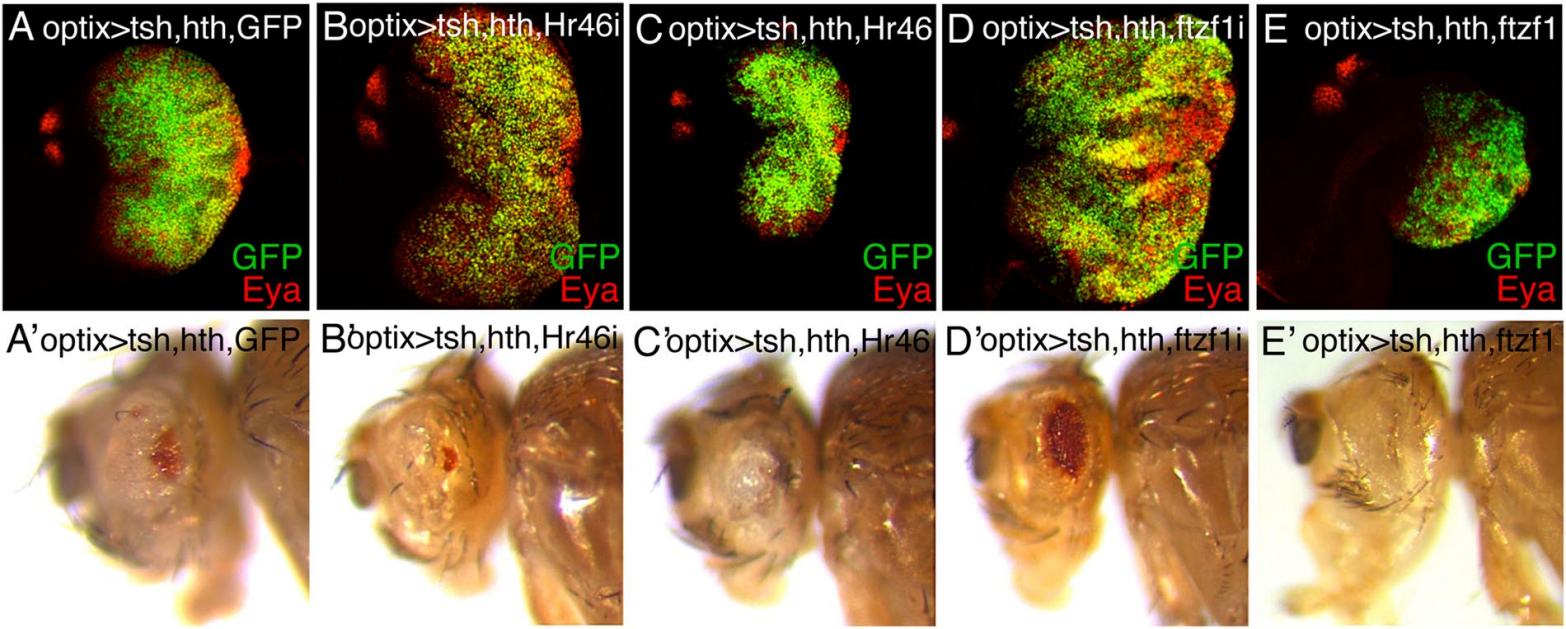
Nuclear receptors *Hr46* and *ftz*-*f1* functionally interact with hth + tsh in inducing tissue overgrowth. L3 eye discs, stained for GFP and Eya (upper panel) and lateral views of adult heads (lower panels) of the indicated genotypes (note that all genotypes harbor equal number of UAS-transgenes). RNAi-mediated attenuation (**B**) or overexpression (**C**) of *Hr46* enhances or suppresses, respectively, the hth + tsh-induced eye disc overgrowth. In adults, however, while Hr46 attenuation enhances the tissue overgrowth/loss of eye (**B’**), its overexpression reduces the tissue overgrowth, but without rescuing retina differentiation (**C’**). RNAi-mediated attenuation of *ftz*-*f1* (**D**) or overexpression (**E**) enhances or suppresses, respectively, the hth + tsh-induced eye disc overgrowth. In this case, *ftz*-*f1* attenuation partly rescues the eye reduction of hth + tsh individuals (**D’**). Co-overexpression of *ftz*-*f1* suppresses the lateral cuticle overgrowth, without rescuing retina differentiation (**E’**).

To test whether differentially expressed genes in the EcR pathway genes participated in controlling the hth + tsh induced overgrowth, we altered the expression levels of *EcR*, *Hr46*/*DHR3*, *ftz*-*f1* and *Blimp*-*1* in the *optix* > *GFP*:*hth* + *tsh* background, either through double-stranded RNAi-specific knock-downs, dominant negative forms (in the case of EcR) or by overexpression. When available, we used several different RNAis per gene (Suppl. Table 3). To evaluate whether varying the expression levels of a gene enhanced or suppressed the hth + tsh-driven phenotype, we took into consideration changes in size and extent of differentiation in eye discs and, in adults, we assessed retina size and amount of undifferentiated cuticle. In these experiments, we found strong interactions with *EcR*, *Hr46*/*DHR3* and *ftz*-*f1* (Figs 5 and 6 and Suppl. Fig. 7).

On its own, overexpression of *EcR* (*optix* > *EcRB1*) did not result in any abnormality (Suppl. Fig. 6). However, when EcR was co-overexpressed with hth + tsh (*optix* > *hth* + *tsh* + *EcR* ^*B1*^) the overgrowth of adult cuticle was exacerbated dramatically (Fig. 5A,C). This was also observed in the eye discs, with co-overexpression of *EcR* further increasing the overgrowth of the tissue (Fig. 5B,D). Co-overexpression of a dominant-negative form of the same receptor (*optix* > *hth* + *tsh* + *EcRB1* ^*W650A*^) caused adult lethality, so we analyzed the effects only on eye discs. *optix* > *EcRB1* ^*W650A*^ discs exhibited moderately reduced retinal differentiation and eye disc size (46% smaller that *optix* > *GFP* control discs). The overgrowth in *optix* > *hth* + *tsh* + *EcRB1* ^*W650A*^ discs was suppressed compared with *optix* > *hth* + *tsh* discs in a similar degree (*optix* > *hth* + *tsh* + *EcRB1* ^*W650A*^ discs were 42% smaller tan *optix* > *hth* + *tsh discs*) (Fig. 5E,F and Suppl. Fig. 8). This set of results indicates that EcR, the expression of which is increased in hth + tsh cells, contributes positively to the hth + tsh-driven tissue overgrowth.

When *Hr46* (Fig. 6A,B) or *ftz*-*f1* (Fig. 6A,D) were attenuated using RNAi, the *optix* > *hth* + *tsh* disc overgrowths were exacerbated. In the case of *Hr46*/*DHR3* and *ftz*-*f1*, it is important to note that neither of the RNAis assayed against either of the two genes produced any significant phenotypic alteration on their own (Suppl. Fig. 6). However, the disc phenotypes were not identical: while in *optix* > *hth* + *tsh* + *Hr46*-*RNAi* the portion of differentiating retina (marked by Eya-only cells) was almost totally obliterated (Fig. 6A’,B’), in *optix* > *hth* + *tsh* + *ftz*-*f1*-*RNAi* there was a moderate, but consistent rescue of the Eya-expressing retina (Fig. 6A’,D’). The co-overexpression of *Hr46* or *ftz*-*f1* produced the opposite effects: a clear reduction of the disc size (Fig. 6A,C,E) and a total obliteration of the retina. This obliteration could derive, in part, from the fact that the expression of *Hr46* (UAS-DHR3 RB) or *ftz*-*f1* (UAS-βftz-f1) on their own resulted in approximately 40% and 60% reduction in adult eye size, respectively (Suppl. Fig. 6). In all, these experiments proved that *EcR*, *Hr46* and *ftz*-*f1*, which are regulated transcriptionally by *hth* + *tsh*, were functionally required for their synergistic effect on growth.

**Figure 7:**
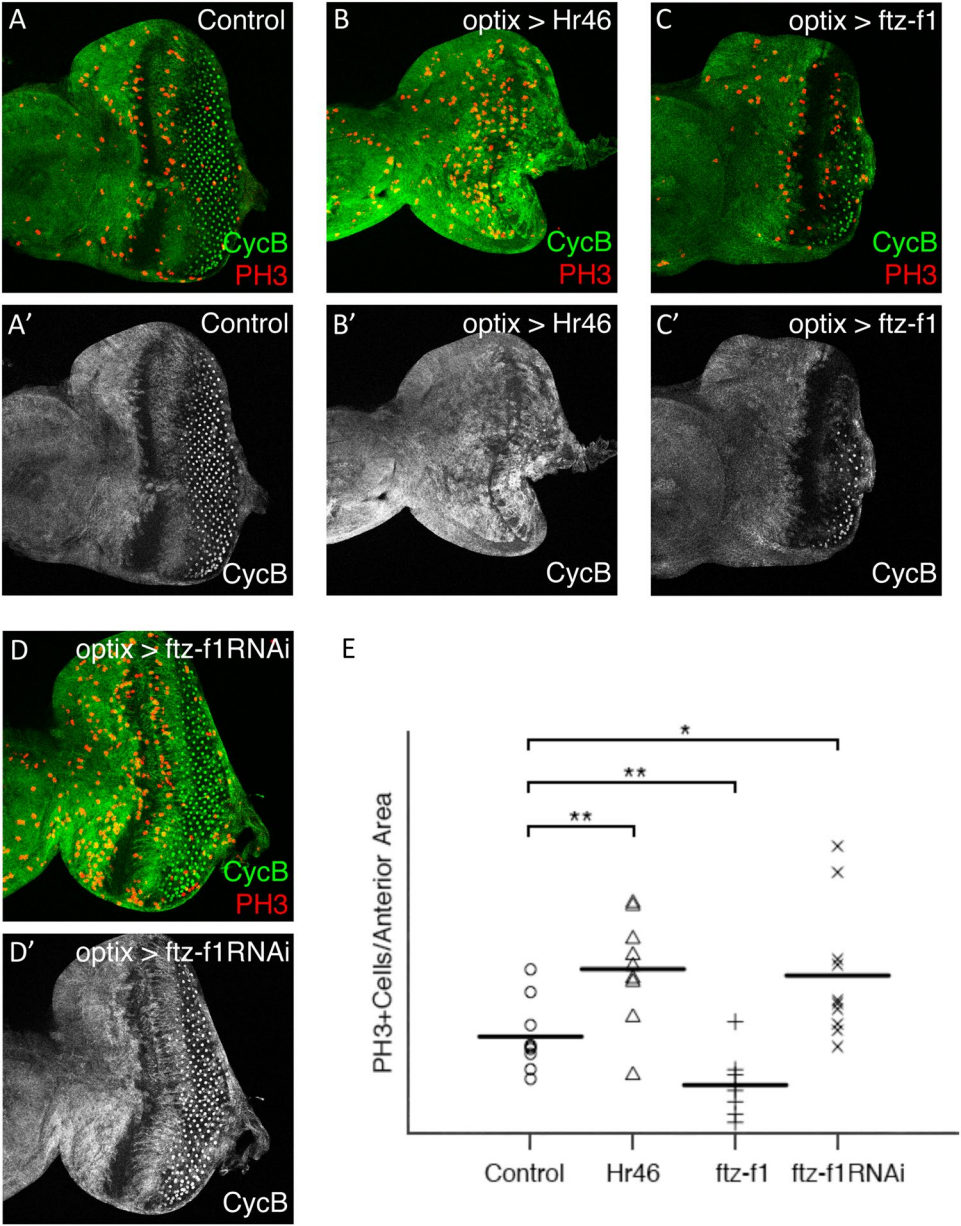
Altering *Hr46* and *ftz*-*f1* expression regulates proliferation of eye progenitors. L3 eye discs of the indicated genotypes (**A**–**D**) stained for cyclinB (cycB, green) and the mitotic marker PH3 (red). Merged and cycB signals are shown. Control discs are *optix*>+. PH3-positive cells were counted in the anterior region of the eye disc, where undifferentiated progenitors reside (outlined in white in **A**). In (**A’**) the double-headed arrow marks the width of the G1-arrested domain (see text for details). (**E**) Statistical analysis of the mitotic density (PH3 + cells/anterior area) indicates that overexpression of Hr46 and RNAi-mediated attenuation of *ftz*-*f1* result in increased proliferation. Note that in both genotypes the G1 arrested domain is narrower than in the control (especially for *optix* > *Hr46*; **B**). On the contrary, overexpression of *ftz*-*f1* results in reduced proliferation.

In addition, our transcriptomic/bioinformatics analysis suggested that some, or all of these nuclear receptors might be exerting their function through the regulation of cell cycle genes. This implied that *EcR*, *Hr46* and/or *ftz*-*f1* should have the potential to regulate the proliferation rates of cells anterior to the MF, where *hth* and *tsh* are normally coexpressed. Recent work indicates that indeed ecdysone is required for the proliferation of imaginal discs^47^, supporting this notion. To test specifically if either *Hr46* or *ftz*-*f1* affect proliferation, we monitored the expression of the G2/mitotic cyclin *cyclin*-*B* (cycB) and the mitotic rate (using the mitotic marker phospho-Histone H3, PH3) of undifferentiated cells in *optix* > *Hr46*, *optix* > *ftz*-*f1* and *optix* > *ftz*-*f1*-*RNAi* (Fig. 7). In control discs (*optix* > *GFP*), proliferation is patterned: it is mostly restricted to progenitors, located at the far anterior of the disc, which express CycB and are mitotically active. Closer to the MF, cells stall their cell cycle transiently in G1, so they lose CycB and do not undergo mitosis (Fig. 7A). In *optix* > *Hr46* discs, though, the density of mitotic (PH3-positive) cells increased dramatically and the CycB gap anterior to the MF narrowed or disappeared, indicating an increased and continuous proliferation (Fig. 7B,E). Next, we tested *ftz*-*f1*. Overexpression of *ftz*-*f1* (*optix* > *ftz*-*f1*) resulted in a strong decrease in the density of PH3 cells in the anterior disc and a widening of the cycB gap anterior to the MF (Fig. 7C,E). While in the contrary experiment, *ftz*-*f1* attenuation (*optix* > *ftz*-*f1*-*RNAi*) increased anterior proliferation, and the cycB gap narrowed (Fig. 7D,E). These results indicate that both *Hr46* and *ftz*-*f1* have the potential to act as cell cycle regulators during eye disc development, and that they have opposing effects on proliferation.

### Hr46/DHR3 and *ftz*-*f1* are regulated by Hth + Tsh

**Figure 8:**
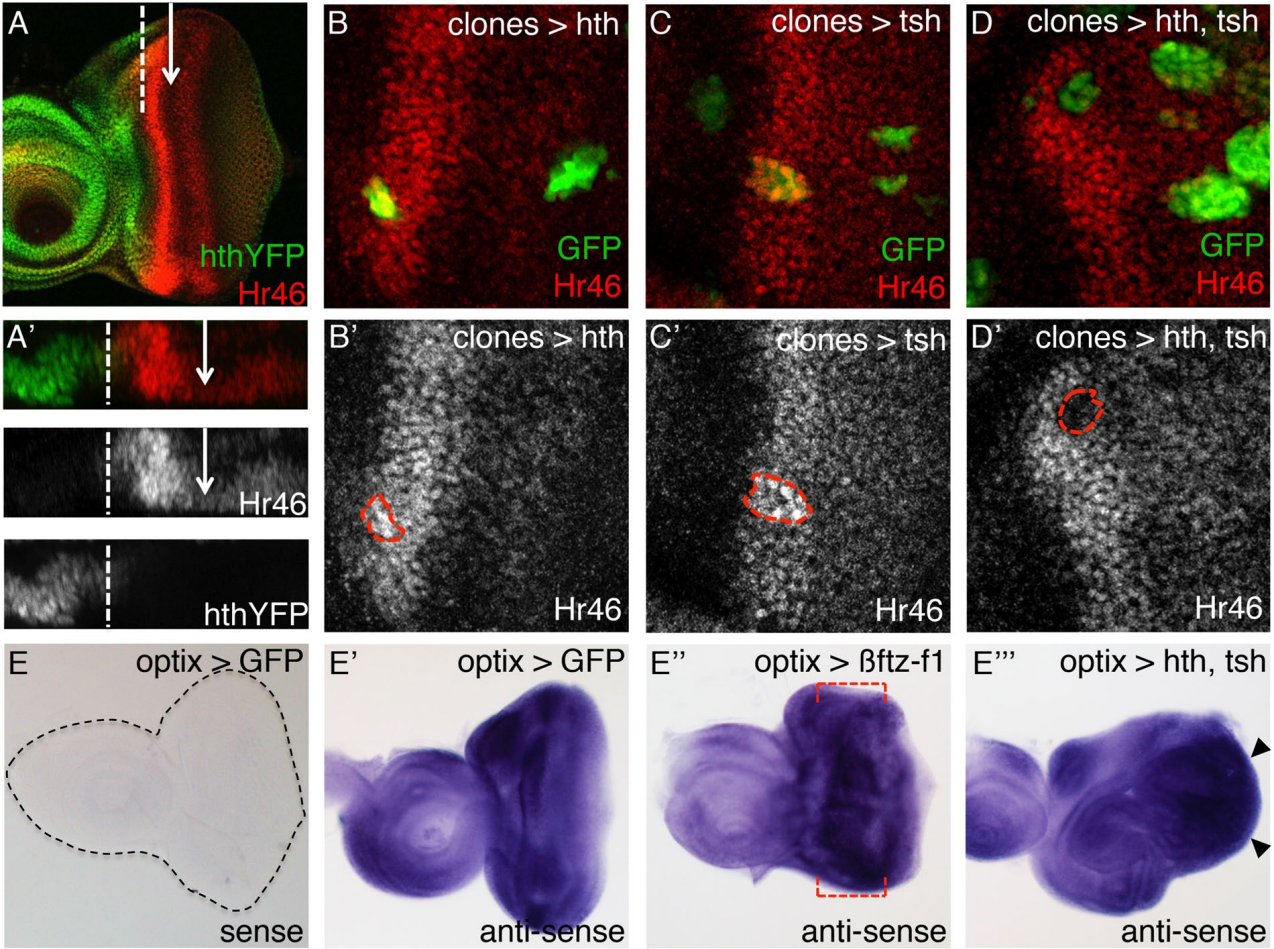
The expression domains of *hth* and *Hr46* are complementary and co-expression of hth + tsh repress *Hr46*. hth:YFP late L3 disc stained with anti-Hr46 (**A**) and the corresponding optical cross-section (**A’**). The arrow marks the morphogenetic furrow (MF) and the dashed line marks the boundary between Hth and Hr46 expression. Clones overexpressing *hth* (**B**,**B’**), *tsh* (**C**,**C’**) or both (**D**,**D’**), marked by GFP (and outlined with the red dashed line), were induced in the eye imaginal disc at 48–72 hours after egg laying. Discs are stained with anti-Hr46. (**E**–**E”’**) *in situ* hybridization with *ftz*-*f1* anti-sense probe in third-instar eye discs from optix > GFP (**E’**), optix > ßfyz-f1 (**E”**) and optix > hth.tsh (**E”’**) larvae. *ftz*-*f1* sense probe was used as a control in optix > GFP eye discs (black dashed line outlines the disc) (**E**). *ftz*-*f1* is transcribed in control eye discs in a dynamic pattern, with high expression in the anterior region and lower levels in the most posterior region. Red dashed lines mark the region where ftz-f1 expression is expected to be higher in optix > ßfyz-f1 discs. hth + tsh discs show higher ftz-f1 levels (black arrowheads mark the regions with especially strong *ftz*-*f1* transcription).

So far, four sets of results indicated that Hr46 could be one of the key players in the response of cells to the combined expression of *hth* and *tsh*: (1) its transcription was specifically downregulated in hth + tsh discs; (2) potential binding sites for Hr46 were found enriched in CREs linked to differentially expressed genes characterized as cell cycle regulators; (3) Hr46 functionally interacted with hth + tsh and showed the capacity to regulate progenitor proliferation; and (4) Hth-binding plus FAIRE-seq data suggested that *Hr46* was a Hth direct target. If *Hr46* regulation were direct, and taking into account that globally *Hr46* was downregulated by hth + tsh, we expected *Hr46* to be repressed by hth + tsh in a cell-autonomous manner. First, we characterized *Hr46* expression during third larval stage to, then, check the effect of hth + tsh expressing clones on its expression. During the third (and last) larval period (L3), the expression of *Hr46*, monitored with an anti-Hr46 antiserum, builds up (Suppl. Figure 9). During early L3, *Hr46* is expressed weakly and ubiquitously in the eye disc. As differentiation moves across the disc, *Hr46* levels increase straddling the MF, peaking anterior to it. This expression is in agreement with ecdysone signaling being active in this region of the disc during L3 (Fig. 3 and ref. 51). Notably, its expression is exclusive to that of Hth. Only at the L3-pupal transition, Hr46 levels raise uniformly throughout the disc, coinciding with the ecdysone pulse that triggers this molting (Suppl. Figure 9). Therefore, during most of the retinal differentiation period, the expression of *hth* and *Hr46* is complementary (Fig. 8A). Since *tsh* expression overlaps *hth* in this anterior region of the eye primordium^10^, the complementarity between *hth* and *Hr46* was consistent with *Hr46* being repressed by hth + tsh. To test this point, we induced cell clones, marked with GFP, expressing either hth or tsh alone, or hth + tsh. Only hth + tsh clones reduced the levels of Hr46, and did so in a cell-autonomous manner (Fig. 8B–D), which agrees with a direct regulation of *Hr46* by hth + tsh.

Another NR differentially transcribed in hth + tsh cells and showing functional interactions with these two transcription factors is *ftz*-*f1*. Using *in situ* hybridization with a probe common to all *ftz*-*f1* isoforms (Fig. 8E–E”’), we detected ftz-f1 transcription in the anterior portion of the eye disc (Fig. 8E,E’), where undifferentiated cells reside. Signal in the differentiated eye is lower. To test that this expression is specific, we hybridized also *optix* > *ftz*-*f1* discs and found that, as expected, ftz-f1 transcription was enhanced in the *optix* domain –i.e. anterior disc (Fig. 8E”). When hth + tsh expression is maintained in *optix* > *hth* + *tsh*, *ftt*-*f1* transcription was substantially increased in the overgrowing tissue (Fig. 8E”’), confirming that hth + tsh can enhance *ftz*-*f1* transcription.

### Potential significance of the hth + tsh control of nuclear receptors in human cancers

The over-proliferative phenotype caused by co-expression of *hth* and *tsh* in the *Drosophila* eye prompted us to ask whether co-ocurrence of their homologues, MEIS and TSHZ, could also be detected in human tumors. A role for MEIS1 in hematopoietic and solid tumors is established, but a potential relationship with TSHZ genes had not been previously explored. Analysis of 116 public datasets on the R2 Genomics Analysis and Visualization Platform (http://r2.amc.nl), representing most major tumor types, showed that MEIS1 and TSHZ1–3 are widely expressed in solid tumors (Suppl. Table 4A). In addition, significant positive correlation between MEIS1 and TSHZ1, TSHZ2, *or* TSHZ3 mRNA expression was found in several major solid tumor types (in 46, 56, and 60 of 91 datasets, respectively; Suppl. Tables 4B and 5). Oncomine analysis of these tumor types datasets showed that MEIS1 and TSHZ1–3 often have mRNA co-overexpression and/or DNA copy number gain in cancer versus normal tissue (Suppl. Tables 4C and 6). Suppl. Figure 10 shows examples for MEIS1 and TSHZ2 in breast and colon cancer with R2 (A,B) and Oncomine (C–F). These data suggest that the synergistic growth-inducing capacity of *hth* and *tsh* in *Drosophila* has a parallel in oncogenic coordinated over-expression of MEIS1 and TSHZ genes in human cancer.

Our global characterization of the *hth* + *tsh*-induced overgrowths in *Drosophila* pointed to a crucial role for three nuclear receptors, *EcR*, *Hr46* and *ftz*-*f1*. In addition, previous work had shown that *hth* and *tsh* directly interact with *yki*, the co-activator of the Salvador-Warts-Hippo pathway, and that this interaction was necessary for the pro-proliferative action of this TF combination^11, 59^. We therefore searched for similar expression signatures in human tumors with MEIS1 and TSHZ co-overexpression on the R2 Platform. We found significant and consistent correlations between MEIS1 and RORA, NR5A2, or YAP1 (the human *Hr46*, *ftz*-*f1*, and *yki* homologues, respectively) in major solid tumor types like breast, colon, and lung cancer (Suppl. Table 7, see also Suppl. Table 5). We had expected a *negative* correlation for RORA, as Hr46 showed a clear repression in hth + tsh cells. However, this difference may be explained either by differences in the “tumoral stage” between the *Drosophila* overgrowths and human tumors -for instance, the *Drosophila* hth + tsh overgrowths are not metastatic (not shown)- or by species-specific differences in the mechanisms driving overproliferation. When globally considered, our results identify a parallelism between the progenitor proliferation program controlled by hth + tsh and the MEIS1/TSHZ-associated oncogenic program.

## Discussion

During organ development and tissue homeostasis cell numbers are tightly controlled. This control is exerted on progenitors, the main proliferating cell population, through their expression of transcription factors which, forming regulatory networks, also preserve them in an undifferentiated state. In this paper we have investigated in *Drosophila* how the input of two conserved transcription factors, which are part of the eye gene network, *hth* and *tsh*, regulate proliferation of eye progenitors.

Integrated analysis of the transcriptome, chromatin accessibility and TF binding motif enrichment data from hth + tsh-induced overgrowths revealed a transcriptional network related to the EcR (Ecdysone Receptor) pathway. At the molting periods, pulses of the active form of the ecdysone hormone, 20-hydroxiecdysone, the major estrogen hormone in insects, trigger the response of the EcR pathway. This response is characterized by stereotypic, staggered expression changes in nuclear receptors that regulate the entry into metamorphosis^46^. Specifically, the pattern of nuclear receptor gene expression in hth + tsh cells, characterized by high *EcR*/*ftz*-*f1* and low *Hr46*/*Blimp*-*1* is typical of a low/moderate ecdysone signaling^47^. During eye development in *Manduca sexta*, moderate levels of ecdysone are required for stimulation of eye proliferation during larval stages. However, low levels of ecdysone arrest cells in the G2 phase, while the high pulse of ecdysone released later during development is responsible for cell cycle exit^60, 61^. A similar situation might be happening during *Drosophila* eye development. In this case, forced maintenance of hth + tsh might induce cell proliferation through the maintenance of a moderate activity of the ecdysone pathway. Four sets of facts strongly suggest that the high EcR/ftz-f1-low Hr46/Blimp-1 pattern of nuclear receptors is instrumental in triggering the hth + tsh-mediated tissue overgrowth. First, modulating the expression or activity of *EcR*, *Hr46* or *ftz*-*f1* affects the hth + tsh-induced overgrowths in non-additive ways; second, changes in nuclear receptors are paralleled by increased expression of cell cycle genes; third, CREs linked to these cell cycle genes show an enrichment of Hr46/ftz-f1-type DNA binding motifs, pointing to a direct regulatory linkage; and fourth, we have observed that *Hr46* and *ftz*-*f1* have the potential to regulate progenitor proliferation. Therefore, we posit that overexpression of hth + tsh results in a specific pattern of nuclear receptor transcription. Part of this may stem from a potential direct regulation by Hth and Tsh of genes such as *Hr46*. Then, expression changes along the nuclear receptor cascade would affect a large number of cell cycle-related genes, which show significant association of nuclear receptors DNA-binding motifs to their CREs, thus leading to sustained tissue growth. We have noted a discrepancy between the direction of the functional interactions of *Hr46* and *ftz*-*f1* with hth + tsh, and the capacity to enhance (Hr46) or decrease (ftz-f1) cell proliferation when assayed individually. For example, co-overexpression of *Hr46* suppresses the hth + tsh overgrowth (Fig. 6), suggesting an anti-proliferative role. However, overexpression of *Hr46* alone *increases* proliferation rates. We do not have an explanation for this discrepancy. However, the EcR pathway is very complex, with nested temporally delayed feedbacks. With this complexity, it is difficult a priori to predict the direction of the interactions. In addition, although not measured in this study, variations in the rate of apoptosis may impact final organ size. Still, we believe the we present solid evidence indicating that hth + tsh promote a specific pattern of nuclear receptor expression; that these nuclear receptors functionally interact with hth + tsh in modulating the overgrowth these TFs induce in progenitor-like cells and that *Hr46* and *ftz*-*f1* are capable of modulating the proliferative pace of undifferentiated progenitors. Similar discrepancies had been described in other model systems. While it has been shown that increased expression of RORβ, one of the *Hr46* homologues, in rat retinal progenitor cells results in an increase in the number of large cell clones^62^, RORα is normally down-regulated or hypo-activated in breast cancer cells (reviewed in ref. 63).

We have noticed within transcriptional profiling data of Yki overexpressing wing primordia, reported in Suppl. Table 4 by Oh and co-workers^64^, a similar signature of differential expression of nuclear receptors as the one we find in *hth* + *tsh* overexpressing eye discs. This similarity may stem from the fact that, in the eye, Hth and Tsh have been shown to be direct partners of Yki^11^, the transcriptional coactivator of the Hippo tumor suppressor pathway^65^. In an epithelial cancer model in the *Drosophila* eye disc characterized by loss of function of *scribbled* (an apico-basal cell polarity regulator) and overexpression of *abrupt* (a BTB-zinc finger transcription factor), a similar pattern of expression was observed, with reduced levels of *Hr46* and *Blimp*-*1* and high levels of *ftz*-*f1* (in this case *EcR* levels were not affected)^66^. Interestingly, ChIP-seq data analysis showed that Abrupt is able to directly regulate *Hr46*, *Blimp*-*1*, *ftz*-*f1* and *EcR* ^66^. A similar repression of ecdysone response genes has been also described in the *Drosophila* ovary, where *abrupt* interacts with *taiman*, a steroid hormone receptor co-activator^67^. In our work, *abrupt* expression levels were up-regulated in eye discs where there was forced maintenance of hth or hth + tsh (with a fold change of approximately 2 in both situations), suggesting also a role in the control of the expression of nuclear receptors in progenitors. More recently, overexpression of *taiman* and *ftz*-*f1* was also shown to be present in a model of invasive cancer driven by RAS in the eye disc^68^. In the cancer models mentioned above, a role for the Hippo pathway has been described^66, 68^. Therefore, a similar nuclear receptor (and probably *abrupt*) expression pattern might be a general feature of Hippo-related tissue overgrowth. Whether this is also the case in human tumors where components of the Hippo-YAP pathway are mutant needs to be investigated.

One interesting aspect of the global regulatory response elicited jointly by *hth* and *tsh* is that this response is quantitative, not qualitative. That is, expression of hth + tsh drives the transcriptional upregulation of many genes but with minor changes in the profile of their CRE activity, as measured by open chromatin profiling. This suggests that hth + tsh operate through CREs that are already active (i.e., open chromatin), rather than by inducing the de novo “opening” of new ones. This behavior contrasts with results analyzing the transcriptional response and CRE activity profiles in eye tumors in the *rasV12*/*scrib* model. Here, the transcriptional changes were paralleled by qualitative changes in CRE activity, with the *de novo* opening of hundreds of promoters and enhancers^57^. This fact seems related to the different nature of the tissue overgrowths in each of the two genotypes. While hth + tsh expression drives continuous proliferation of progenitors (i.e. hyperplastic growth), *rasV12*/*scrib* tissues are metastatic.

The dual control of cell fate and proliferation makes organ specification TFs a “vulnerable link”. Particularly, it is often the case that mutations affecting the expression of an organ- or cell-type selector TF result in cancer developing from this same organ. Examples of this are the eye and pancreas TF Pax6 in retinoblastoma and pancreatic cancer^69, 70^; myogenic MyoD1 in rhabdomyosarcoma^71^; hematopoietic progenitor TFs MEIS1 and TAL1 in leukemia^72, 73^; neural crest SOX10 and MITF in melanoma^74, 75^ or GATA3 in breast cancer^76^. And more generally, many cancer driver mutations affect TFs^77^. In particular, the oncogenic role of MEIS1 has been documented. Here, we have established for the first time that MEIS1 and TSHZ occur in coordinated over-expression in several major solid tumors types, an association that may recapitulate the functional synergism of *hth* and *tsh* in the fly eye primordium.

Co-overexpression of hth + tsh results in transcriptional changes and functional interactions that bear similarity with those observed in tumors where Estrogen Receptor alpha (ERα) and NR5A2/LRH-1 (*ftz*-*f1* homologue) play important roles, such as breast cancer^78–80^. Interestingly, it has been reported that NR5A2 expression is also increased in pancreatic cancer (see also Suppl. Tables 5 and 7) where it promotes cell growth through stimulation of major cell cycle regulators cyclin D1, cyclin E1 and c-Myc^81^, and that NR5A2/LRH-1 represses the cell cycle inhibitor p21^82^. In addition, through ChIP-seq and gene expression experiments, the estrogen receptor (and other nuclear receptors, such as the androgen receptor) has been shown to directly regulate genes involved in cell cycle progression^83–86^. The similarities between the *Drosophila* mechanism described here and the expression correlations found in human tumors suggest a scenario where MEIS1 and TSHZ genes, if co-overexpressed, might be driving transformation through the regulation of nuclear receptors which, then, would be translated into a general regulatory effect on many cell cycle-related genes.

## Materials and Methods

### GEO accession numbers

GSE65252 contains two series: RNA-seq (GSE65250) and FAIRE-seq (GSE65251) data.

### Fly strains and genetic manipulations

All crosses were set up and raised at 25 °C under standard conditions. We used the UAS/GAL4 system for targeted misexpression^87^. The following stocks were used: *optix2*.*3*-*GAL4* (gift from R. Chen, Baylor College of Medicine); *UAS*-*GFP* ^88^; *UAS*-*131*-*GFPhth* ^89^; *UAS*-*Flag*-*HA*-*tsh* (gift from C. M. Luque, Universidad Autónoma, Madrid, Spain); *yw*, hs-*FLP* ^122^; *act* > *y* + > Gal4^90^ with a recombined UAS-*GFP* transgene; Hth-YFP (CPTI-001356; Flannotator); EcRE-lacZ (Bloomington #4517). Fly stocks used for the screen are listed in Suppl. Table 3. *optix* > *GFP*:*hth* and *optix* > *tsh* larvae were collected from *optix2*.*3*-*GAL4* to *UAS*-*GFP*:*hth* or *UAS*-*HA*:*tsh* crosses. For RNA-seq and FAIRE-seq experiments, *optix* > *GFP*:*hth* + *tsh* larvae were obtained directly from an *optix2*.*3*-*GAL4*,*UAS*-*Flag*-*HA*-*tsh*;*UAS*-*131*-*GFPhth*/*SM6^TM6B* stock (“hth + tsh_stock”; biological replicate #1) or derived from the cross of *optix2*.*3*-*GAL4*,*UAS*-*Flag*-*HA*-*tsh*; + /*SM6^TM6B* to *UAS*-*GFP*:*hth* (“hth + tsh_cross”; biological replicate #2). As FAIRE-seq control we used the data sets previously obtained in the laboratory using two reference strains, Oregon-R (wild type) and *FRT82B* (Flybase).

All lines listed in the Suppl. Table 3 (RNAi, overexpression and dominant negative) were crossed to the *optix2*.*3*-*GAL4* driver line and the *optix2*.*3*-*GAL4*,*UAS*-*Flag*-*HA*-*tsh*;*UAS*-*131*-*GFPhth*/*SM6^TM6B* stock. All crosses were maintained at 25 °C. For Hr46 we tested the efficacy of three anti-Hr46 RNAi lines using an anti-Hr46 antiserum (data not shown). In all cases, we detected a reduction of the Hr46 signal, the reduction being most extreme for line 106837. Flies were observed under a LEICA MZ 9.5 stereomicroscope and pictures of heads from adults of each genotype were taken with a LEICA DFC320 digital camera.

Random ectopic expression clones were generated using the flip-out method^90^. *yw*, *hs*-*FLP* ^*122*^; *act* > *y* + > *Gal4*;; *UAS*-*GFP*/*TM6B* females were crossed to *UAS*-*Flag*-*HA*-*tsh*, *UAS*-*131*-*GFPhth* or *UAS*-*Flag*-*HA*-*tsh*;*UAS*-*131*-*GFPhth* males. Clones were induced by heat shock (20 min at 37 °C) between 48 h and 72 h AEL (after egg laying) and then maintained at 25 °C. Clones were positively marked with GFP.

### Immunostaining

Eye-antennal imaginal discs from wandering third instar larvae were dissected and fixed according to standard protocols. Primary antibodies used were: mouse anti-Eya 10H6 at 1:100 (Developmental Studies Hybridoma Bank, DSHB), rabbit anti-ßGal at 1:1000 (Cappel), mouse anti-CycB F2F4 at 1:100 (DSHB), rabbit anti-PH3 at 1:1000 (Sigma), rat anti-ELAV 7EBA10 at 1:1000 (DSHB), rabbit anti-HA 9110 at 1:1000 (Abcam), guinea pig anti-Hth at 1:3000^91^, rabbit anti-Hr46 at 1:50 (gift from Carl S. Thummel, University of Utah School of Medicine), mouse anti-CycA A12 at 1:10 (DSHB), mouse anti-Dacapo NP1 at 1:4 (DSHB), mouse anti-CycE at 1:20 (gift from Helena Richardson). Alexa-Fluor conjugated secondary antibodies and rhodamine phalloidin (R415) were from Molecular Probes. Images were obtained with the Leica SP2 confocal system and processed with Adobe Photoshop.

### RNA probe synthesis

A ftz-f1 digoxigenin (DIG)-labeled RNA probe was synthesized through two PCR reactions using ftz-f1-specific primers with GC-enriched tails (5′‐GGCCGCGGCAGTGGCAATAATGGCAATC‐3′ and 5′‐CCGGCCGCCGATCCTATTCCAGCCTTG‐3′) and universal primers (5′-GAGAATTCTAATACGACTCACTATAGGGCCGCGG-3′ and 5′-AGGGATCCTAATACGACTCACTATAGGCCCCGGC-3′). PCR products were purified from gel using a Qiagen Kit. Using the DIG RNA Labelling Kit (SP6/T7) from Roche, the RNA probe was synthesized with T7 RNA polymerase. After synthesis, precipitation of the probe was done using LiCl and ethanol. The probe was then ressuspended in DEPC treated water.

### *In situ* hybridization

Larvae were dissected in cold PBS, fixed in 3, 7% paraformaldehyde (in PBS) during 20 minutes and refixed in 3, 7% paraformaldehyde (in PBT) during 20 minutes, always at room temperature. After fixation, discs were washed at room temperature in PBTween (PBS with 0, 1% Tween) during 25 minutes (5 washes of 5 minutes each), in PBTween:Hybridization solution (1:1) during 5 minutes and in hybridization solution during 5 minutes. Hybridization solution (HS) contains formamide, 20xSSC, salmon sperm DNA 10 mg/ml, Tween20 (10%) in DEPC treated water. Then, discs were prehybridized for 1 hour at 60 °C in HS. The hybridization reaction was carried out overnight at 60 °C with a hybridization mix (HS, single stranded DNA (4, 6 mg/mL) and labeled probe). After incubation, discs were washed in HS during 20 minutes at 60 °C, in HS:PBT (1:1) during 20 minutes at 60 °C and in PBT during 1 hour (3 washes of 20 minutes each) at room temperature. Afterwards, discs were incubated for 2 hours at room temperature with anti-digoxigenin antibody coupled to alkaline phosphatase (Roche Diagnostics) diluted 1:1000 in PBT. Excess antibody was removed by washing with PBT (a quick wash and then three washes of 20 minutes each) at room temperature. Discs were then equilibrated for 10 minutes (two washes of five minutes each) at room temperature in AP Buffer (fresh solution containing 1 M Tris pH 9, 5, 1 M MgCl2, 4 M NaCl, Tween20 (10%) in DEPC-treated water). Color development was performed at 37 °C (5 minutes) in AP Buffer containing NBT and BCIP. Staining was stopped with PBT (two washes of 5 minutes each) at room temperature. Afterwards discs were post-fixed in 3, 7% paraformaldehyde in PBTween, washed in PBTween and cleared in ethanol.

### Scanning electron microscopy (SEM)

Female flies were transferred to 75% ethanol and equilibrated for 24 hours at room temperature. Flies were dehydrated through ethanol series (80%, 90%, 95% and twice 100%; 12–24 hours each step). Flies were then air-dried and mounted onto SEM stubs covered with carbon tape and sputter coated with gold (Edwards Six Sputter). Images were obtained using a JEOL 6460LV scanning electron microscope.

### Quantification of PH3^+^ cells

Third instar eye imaginal discs from control, optix > Hr46 (UAS-DHR3RB), optix > αftz-f1, and optix > ftz-f1RNAi (#2959) were stained with anti-PH3 and anti-CycB. The anterior area of the eye disc was defined by creating a surface and the PH3^+^ cells first were automatically identified and then manually curated. Finally, the number of PH3^+^ cells that fall within the created surface were detected. This analysis was made using the IMARIS x64 7.7.2 software. The ratios between the PH3^+^ cells and the anterior area (n = 7–10) were calculated and represented as dots (control), triangles (optix > Hr46), plus (optix > ftz-f1) and cross (optix > ftz-f1RNAi) and the means were represented as horizontal bars. The graphical output was generated using R. Statistical significance was determined using an ANOVA test.

### RNA-Seq

Wandering third instar larvae raised at 25 °C under standard conditions were dissected in PBS to extract 50 eye-antennal imaginal discs. RNA was extracted using the RNAqueous micro kit (Ambion). RNA quality was checked using Agilent RNA 6000 Nano Kit. RNA libraries were prepared for sequencing using a standard Illumina TruSeq protocol. Libraries were validated quantitatively (Qubit) and qualitatively (Agilent DNA 1000 Kit, Agilent Technologies 2100 Bioanalyzer).

### FAIRE-Seq

100 eye-antennal imaginal discs from wandering third instar larvae raised at 25 °C under standard conditions were dissected in PBS. The imaginal discs were first dissected, fixed and lysed. The eye-antennal discs were then uncrosslinked and sonicated. DNA purification was achieved by phenol chloroform extraction (MaXtract High Density Kit). DNA libraries were prepared for sequencing using a standard protocol. Libraries were validated quantitatively (Qubit) and qualitatively (Agilent High Sensitivity DNA Kit, Agilent Technologies 2100 Bioanalyzer).

### RNA-seq and FAIRE-seq reads preprocessing

Reads containing residuals of adapters sequences were discarded (FastX clipper version 0.013 with option -M15). Quality control assessment on the reads was performed using the software FastQC (version 0.9), checking for PHRED quality >20 and different primer contaminations. Reads passing the filtering were mapped against *D*.*melanogaster* FlyBase genome release 5 with TOPHAT v2.0 (default parameters)^92^.

### RNA-seq differential expression analysis

To compute gene expression levels, we performed HTSeq (option str = no)^93^. Only uniquely mapped reads falling in exons based on the species-specific FlyBase annotation *D*.*melanogaster* 5.45 were considered.

Differential expression analysis between HTH + TSH (two replicates) and wt (one replicate) was performed using the Bioconductor package DESeq version 1.10.1 (R version 2.15). For contrasts with no replicates available, such as HTH vs wt and TSH vs wt, we utilized the parameters method = ‘blind’, shareMode = ‘fit-only’ to estimate dispersions across samples. Genes presenting low expression across samples, namely, less than 1RPKM in more than 3 samples were not considered for differential expression analysis.

### FAIRE-seq analysis

Pre-processed reads were mapped against *D*. *melanogaster* reference genome release 5 using Bowtie2^94^. Open chromatin levels were computed as the number of reads mapping within Drosophila pre-defined regions^33^ using HTSeq^93^, parameter (str = no). The set of pre-computed *D*. *melanogaster* regulatory regions are defined by a thorough genome-cut which considers sequence conservation, exon skipping and insulator class I binding^33^.

### FAIRE-seq differential expression

Regions with less than 10RPKM in three samples were excluded for differential open chromatin analysis. Differential open-chromatin was performed as described in the RNA-seq differential expression analysis section. The main difference is that instead of genes we use as features regulatory regions ids. The contrast performed defined differential open regions between HTH + TSH (two replicates) and wild-type (two replicates).

### Association between genes and open-chromatin regions

Peaks were assigned to a gene if they were falling 5-kb upstream of its TSS or either limited by the nearest upstream gene, in its intronic regions or in 5-kb downstream of a gene limited by the closest downstream gene.

### Association between open-chromatin and HTH binding

ChIP for HTH, Sd, Yki transcription factors in late third instar (wandering) larvae has been performed by ref. 59. ChIP locations were translated to regulatory region ids if they were presenting an overlap fraction of 40% (overlapSelect f = 0.4).

To assess whether differential open regions between HTH + TSH and wildtype were associated with HTH binding we compared the log2 fold change of FAIRE regions in bound and not bound HTH regions. Wilcoxon singed-ranked test was performed to assess its statistically significance.

### Co-expression using Pavlidis Template Matching

Pavlidis Template Matching^95^ was used to find genes showing a similar or opposite expression profile of EcR (by starting from EcR expression template, p-value < 0.01).

### Gene Ontology term enrichment

Gene ontology enrichment for different gene sets was computed using the tool FlyMine^96^, whereas ranked lists of genes were inputted in GOrilla^97^.

### Motif enrichment discovery

Motif discovery was performed with the tools i-*cis*target^33^ and its Cytoscape version, iRegulon^98^. In brief it searches for overrepresented motifs in a set of co-expressed genes and across evolution. The following parameters were used: motif collection version 2 (6385 position weight matrices) and region mapping equal to 5 Kb upstream and full transcript. i-*cis*target is a hybrid method that allows finding both known and new motifs. The new motifs are also represented as position weight matrices and are a collection of thousands of “candidate” motifs found by other studies for which the binding factor is yet unknown. This collection includes highly conserved words, but also enriched words discovered in chromatin binding data from ENCODE and modENCODE. In addition, i-*cis*target allows finding motifs from orthologous factors, including yeast, mouse, and human, thereby greatly expanding the number of possible TFs. Finally, the number of *Drosophila* transcription factors without a possible motif is very limited, thanks to recent high-throughput approaches^99^, and the porting of binding motifs from other species^52^.

### Gene Set Enrichment Analysis

We used the tool Gene Set Enrichment Analysis (GSEA)^100^ to assess if open-chromatin regions are enriched in either up or down-regulated genes at the gene expression level (hth + tsh *versus* control). Therefore, we inputted two sets of genes with significantly up or down open-chromatin regions and a ranked list of genes based on the −log p value from the differential expression analysis.

### mRNA expression and DNA copy number analysis in human cancer datasets

Affymetrix datasets of 103 different studies on human cancer types were retrieved from the public Gene Expression Omnibus (GEO) dataset on the National Center for Biotechnology Information (NCBI) website^101^. We selected studies using the Affymetrix Gene Chip Human Genome U133 Plus 2.0 array plafform (Affymetrix, Santa Clara, CA) since this was the most common platform in the database. Also, probes for the *TSHZ1*-*3* genes are not present on earlier versions of the Affymetrix platform. Annotations and clinical data for the datasets analyzed are available through their GEO ID’s (Suppl. Table 3) from http://www.ncbi.nlm.nih.gov/geo/query/. CEL data were downloaded, and analyzed as described^102^. Briefly, gene transcript levels were determined from data image files using GeneChip operating software (MAS5.0 and GCOS1.0, from Affymetrix). Samples were scaled setting the average intensity of the middle 96% of all probe-set signals to a fixed value of 100 for every sample in the dataset, to allow transcript level comparison between micro-arrays and between studies. The TranscriptView genomic analysis and visualization tool (http://bioinfo.amc.uva.nl/human-genetics/transcriptview/) was used to select probe-sets. These had to show unique mapping in an anti-sense position within a 3′ exon and/or the 3′ UTR of the gene. When multiple correct probe-sets were available for a gene, the probe-set with the highest average expression and amount of present calls for that dataset was considered as the best probe-set. These were: 204069_at (*MEIS1*), 210174_at (*NR5A2*), 226682_at (*RORA*), 242385_at (*RORB*), 223283_s_at (*TSHZ1*), 235815_at (*TSHZ2*), 223392_s_at (*TSHZ3*), and 224894_at (*YAP1*). When results of the best probe-set conflicted with other probe-sets for that gene, the data are not presented. Analyses on the GEO datasets Analyses were performed using R2; a genomics analysis and visualization platform developed in the Department of Oncogenomics at the Academic Medical Center, Amsterdam, The Netherlands (http://r2.amc.nl).

### Statistical analysis of of mRNA expression and DNA copy number in human cancer

Correlations between *MEIS1* and other gene mRNA expression in R2 were calculated using a Pearson test on 2 log-transformed expression values (with the significance of a correlation determined by t = R/sqrt((1 − r^2^)/(n − 2)), where R is the correlation value and n is the number of samples, and distribution measure is approximately as t with n − 2 degrees of freedom). The Statistical Package for the Social Sciences software package for Windows (Version 13.0) was used for all statistical analyses. All numerical results are expressed as the mean value ± S.E.M., and *P* < 0.05 was considered significant in all tests. For all tests on Oncomine (http://www.oncomine.org), the website standard settings were used, and values are shown as 2 log-median centered, with statistically significant differences determined by t-testing.

## Electronic supplementary material

Supplementary Information

Supplementary Table 1

Supplementary Table 2

Supplementary Table 3

Supplementary Table 5

Supplementary Table 6

Supplementary Table 7
