# Supplementary material for: Nuclear receptors connect progenitor transcription factors to cell cycle control
Source: Sci Rep. 2017 Jul 7;7:4845. doi: 10.1038/s41598-017-04936-7 (PMC5501803; doi:10.1038/s41598-017-04936-7)
Supplement: Supplementary file 1 — Supplementary Information [file 41598_2017_4936_MOESM1_ESM.pdf]

Supplementary Data

Nuclear receptors connect progenitor transcription factors to cell cycle control.

Marta Neto[1,3], Marina Naval-Sánchez[2], Delphine Potier[2], Paulo S. Pereira[3], Dirk Geerts[4], Stein Aerts[2]*, Fernando Casares[1]*

1- CABD, Andalusian Centre for Developmental Biology, CSIC-UPO-JA, 41013 Seville, SPAIN.
2- School of Medicine, University of Leuven, box 602 3000 Leuven, BELGIUM.
3- IBMC/Instituto de Investigação e Inovação em Saúde, Universidade do Porto, 4200-135 Porto, PORTUGAL.
4- Department of Pediatric Oncology. Erasmus University Medical Center, 3015 CN Rotterdam, THE NETHERLANDS.

(*) Contacts: Stein.Aerts@med.kuleuven.be; fcasfer@upo.es

Contains:

- Supplementary Figures S1-10
- Supplementary Table S4
- Legends to Supplementary data (Figures S1-10; Tables S1-7)

Neto et al., Figure S1

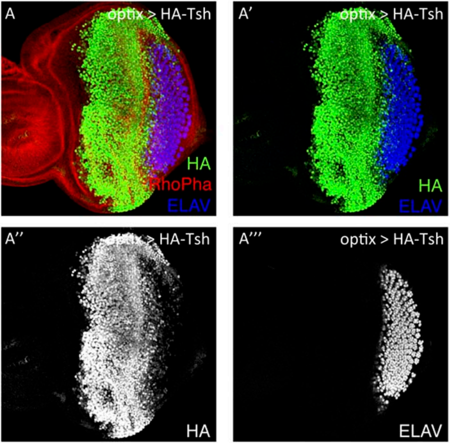

Neto et al., Figure S2

A

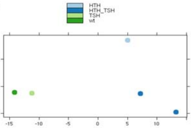

B

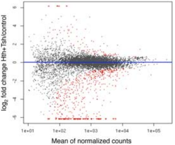

C

Hth+Tsh down-regulated genes

Gene Ontology Term	P-adjusted
Generation of neurons	*2.23E-28*
Nervous system development	*2.96E-21*
Signaling	*6.31E-21*
Cell communication	*1.78E-20*
System development	*3.87E-19*
Axon development	*8.49E-19*
Anatomical structure morphogenesis	*6.06E-18*
Compound eye photoreceptor cell differentiation	*3.40E-17*
Cell projection organization	*9.85E-17*

D

Hth+Tsh up-regulated genes

Gene Ontology Term	P-adjusted
DNA-dependent DNA replication	*5.82E-14*
DNA metabolic process	*1.13E-07*
Nuclear cell cycle DNA replication	*2.93E-07*
Cell cycle	*3.05E-05*
Single organism organelle organization	*8.82E-05*
Mitotic cell cycle process	*0.00048*
Pyrimidine deoxyribonucleotide metabolic process	*0.00137*
Cytoskeleton organization	*0.00191*
Meiotic cell cycle process	*0.00241*

Neto et al., Figure S3

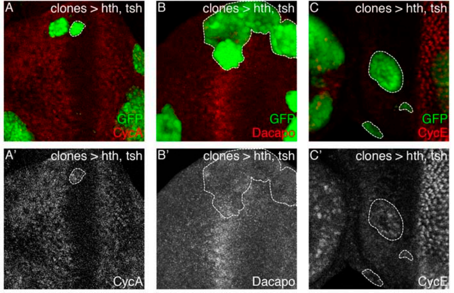

Neto et al., Figure S4

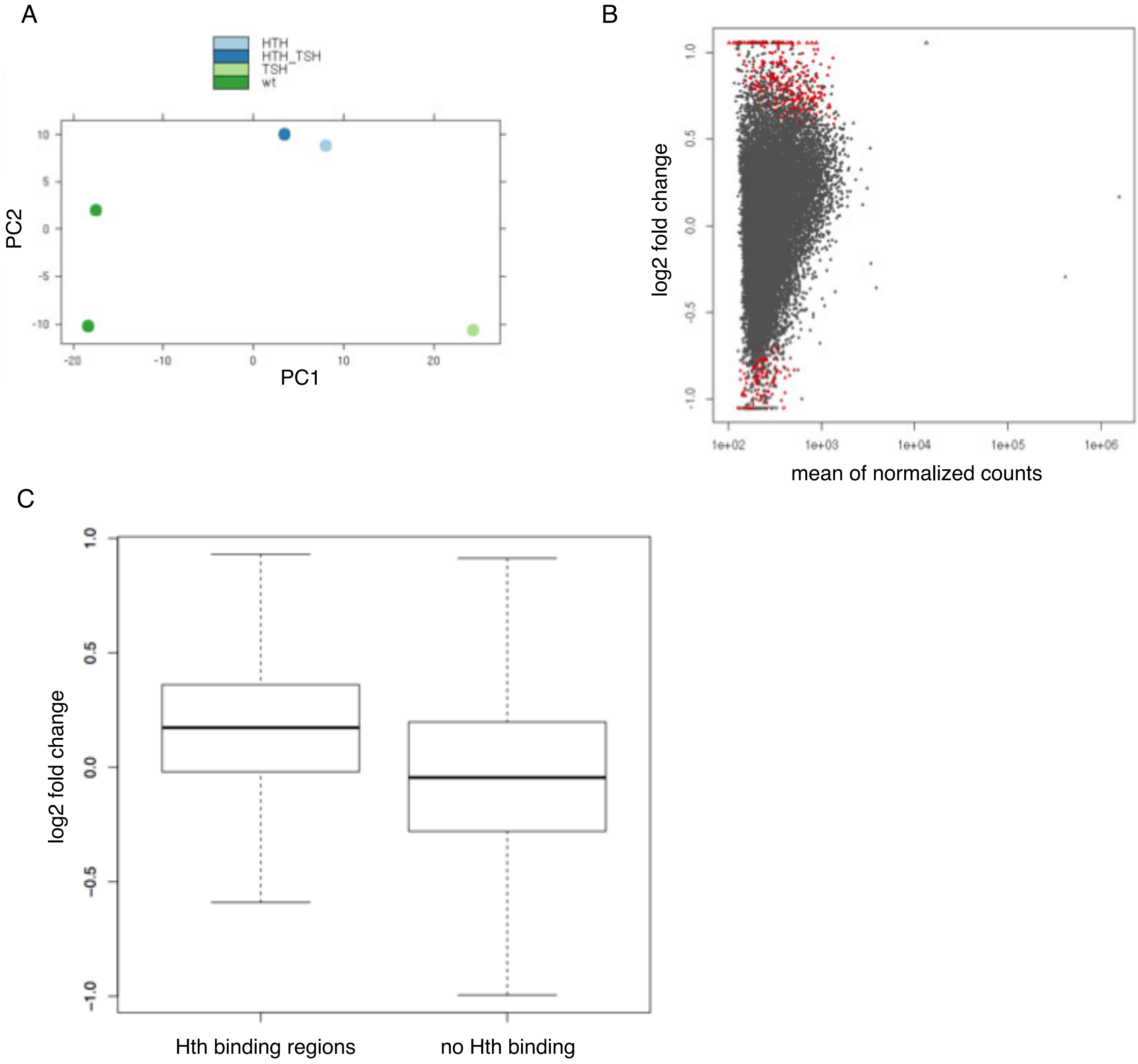

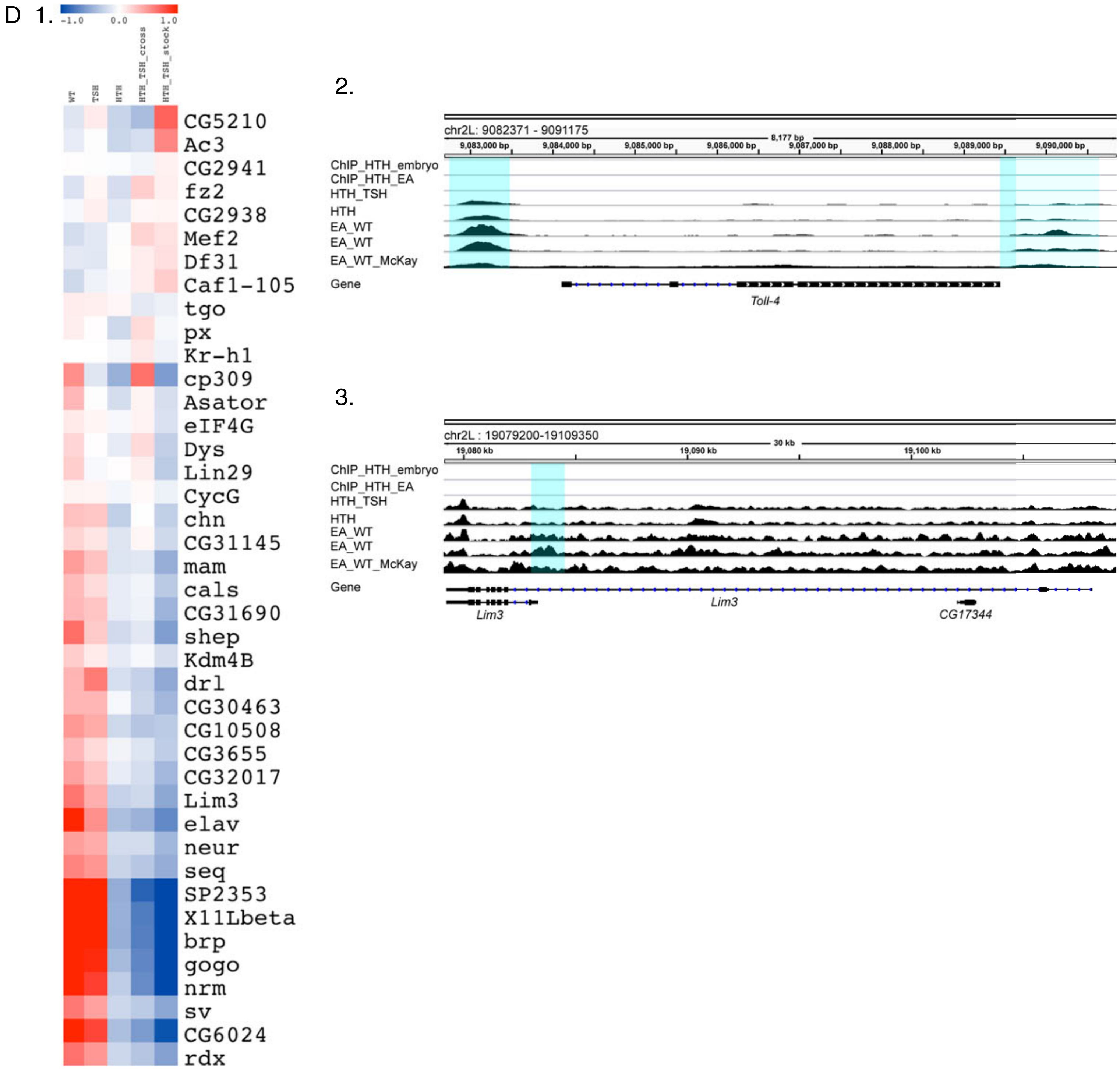
D 1.
-1.0
0.0
1.0
WT
TSH
HTH
HTH_TSH_cross
HTH_TSH_stock
CG5210
Ac3
CG2941
fz2
CG2938
Mef2
Df31
Caf1-105
tgo
px
Kr-h1
cp309
Asator
eIF4G
Dys
Lin29
CycG
chn
CG31145
mam
cals
CG31690
shep
Kdm4B
drl
CG30463
CG10508
CG3655
CG32017
Lim3
elav
neur
seq
SP2353
X11Lbeta
brp
gogo
nrm
sv
CG6024
rdx
2.
chr2L: 9082371 - 9091175
8,177 bp
9,083,000 bp
9,084,000 bp
9,085,000 bp
9,086,000 bp
9,087,000 bp
9,088,000 bp
9,089,000 bp
9,090,000 bp
ChIP_HTH_embryo
ChIP_HTH_EA
HTH_TSH
HTH
EA_WT
EA_WT
EA_WT_McKay
Gene
Toll-4
3.
chr2L : 19079200-19109350
30 kb
19,080 kb
19,090 kb
19,100 kb
ChIP_HTH_embryo
ChIP_HTH_EA
HTH_TSH
HTH
EA_WT
EA_WT
EA_WT_McKay
Gene
Lim3
Lim3
CG17344

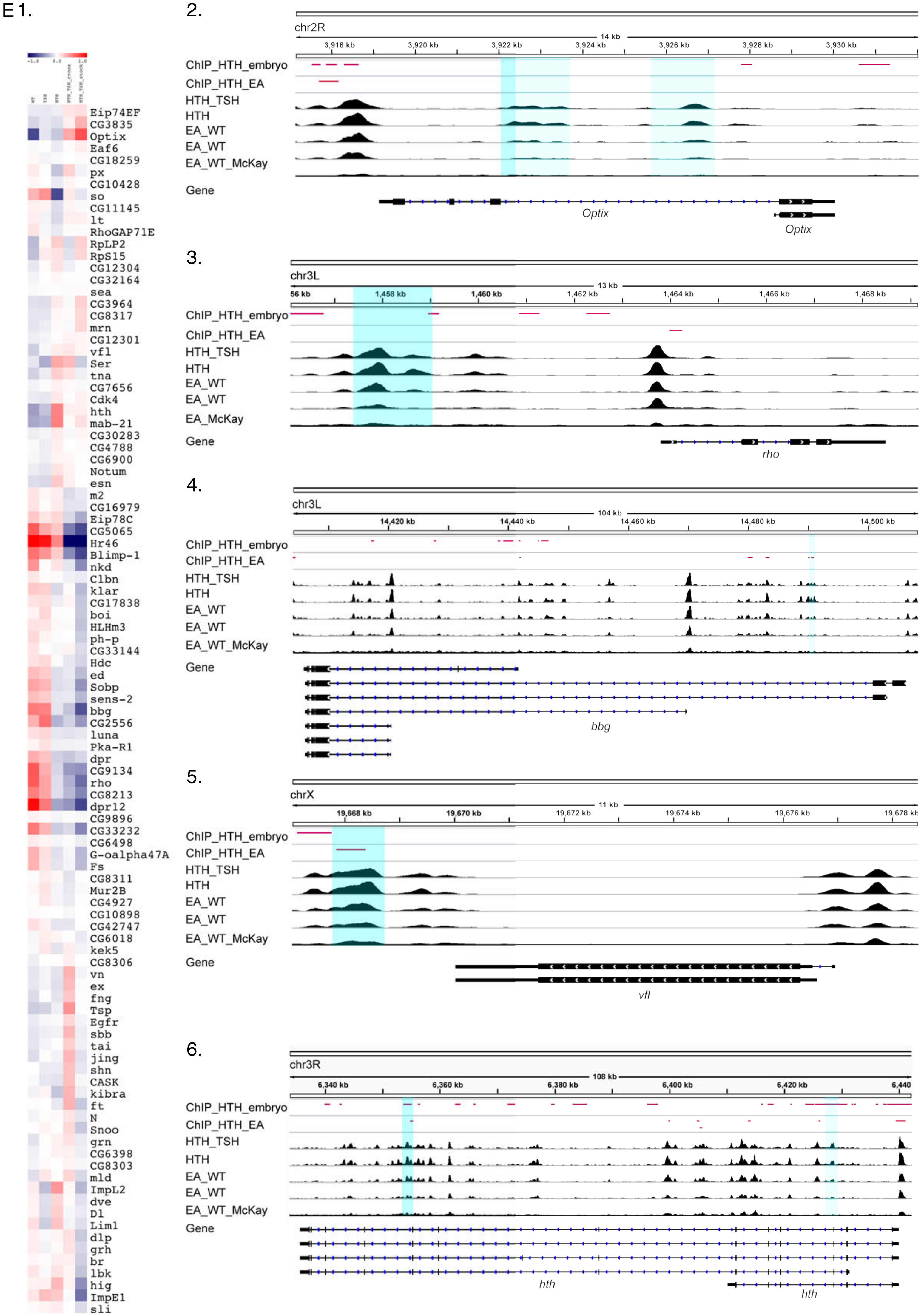
E1.
-1.0
0.0
1.0
Eip74EF
CG3835
Optix
Eaf6
CG18259
px
CG10428
so
CG11145
lt
RhoGAP71E
RpLP2
RpS15
CG12304
CG32164
sea
CG3964
CG8317
mrn
CG12301
vfl
Ser
tna
CG7656
Cdk4
hth
mab-21
CG30283
CG4788
CG6900
Notum
esn
m2
CG16979
Eip78C
CG5065
Hr46
Blimp-1
nkd
Clbn
klar
CG17838
boi
HLHm3
ph-p
CG33144
Hdc
ed
Sobp
sens-2
bbg
CG2556
luna
Pka-R1
dpr
CG9134
rho
CG8213
dpr12
CG9896
CG33232
CG6498
G-oalpha47A
Fs
CG8311
Mur2B
CG4927
CG10898
CG42747
CG6018
kek5
CG8306
vn
ex
fng
Tsp
Egfr
sbb
tai
jing
shn
CASK
kibra
ft
N
Snoo
grn
CG6398
CG8303
mld
ImpL2
dve
Dl
Lim1
dlp
grh
br
lbk
hig
ImpE1
sli
2.
chr2R
14 kb
3,918 kb
3,920 kb
3,922 kb
3,924 kb
3,926 kb
3,928 kb
3,930 kb
ChIP_HTH_embryo
ChIP_HTH_EA
HTH_TSH
HTH
EA_WT
EA_WT
EA_WT_McKay
Gene
Optix
Optix
3.
chr3L
13 kb
56 kb
1,458 kb
1,460 kb
1,462 kb
1,464 kb
1,466 kb
1,468 kb
ChIP_HTH_embryo
ChIP_HTH_EA
HTH_TSH
HTH
EA_WT
EA_WT
EA_McKay
Gene
rho
4.
chr3L
104 kb
14,420 kb
14,440 kb
14,460 kb
14,480 kb
14,500 kb
ChIP_HTH_embryo
ChIP_HTH_EA
HTH_TSH
HTH
EA_WT
EA_WT
EA_WT_McKay
Gene
bbg
5.
chrX
11 kb
19,668 kb
19,670 kb
19,672 kb
19,674 kb
19,676 kb
19,678 kb
ChIP_HTH_embryo
ChIP_HTH_EA
HTH_TSH
HTH
EA_WT
EA_WT
EA_WT_McKay
Gene
vfl
6.
chr3R
108 kb
6,340 kb
6,360 kb
6,380 kb
6,400 kb
6,420 kb
6,440
ChIP_HTH_embryo
ChIP_HTH_EA
HTH_TSH
HTH
EA_WT
EA_WT
EA_WT_McKay
Gene
hth
hth

Neto et al., Figure S5

Cluster Rank	NES	Best motif logo	Best motif name	Candidate TFs
1	7.4	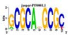	jaspar-PF0001.1	ewg
4	4.9	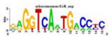	selexconsensus-EcR_usp	EcR
6	4.8	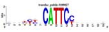	transfac_public-M00027	sd
9	4.4	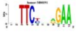	homer-M00191	Stat92E
18	3.9	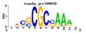	transfac_pro-M00920	E2f, E2f2, Dp
21	3.8	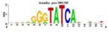	transfac_pro-M01345	so, Optix

+bcd + ATF3 +Adf1 +slbo +oc +MAd +DNApoliota +gem + pnr

Neto et al., Figure S6

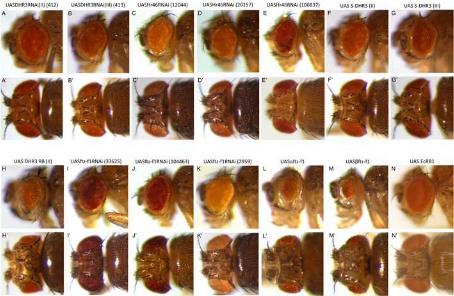

Neto et al., Figure S7

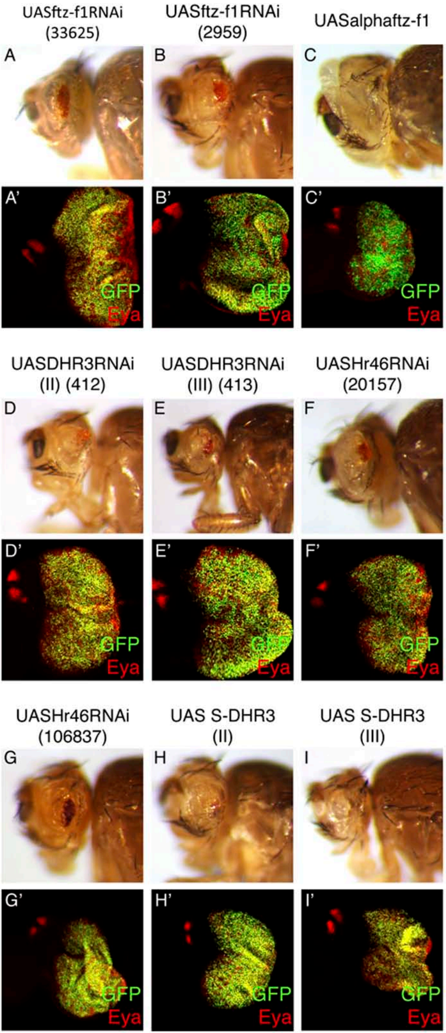

Neto et al., Figure S8

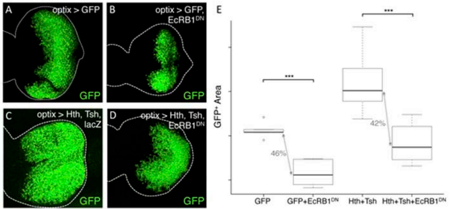

Neto et al., Figure S9

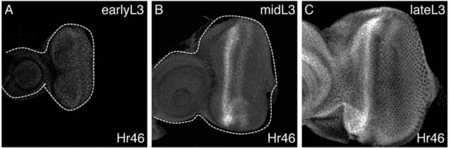

Neto et al., Figure S10

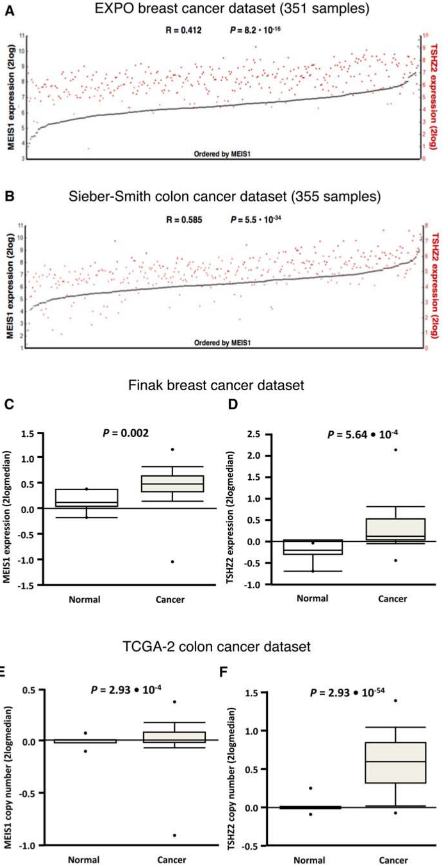

Figure Legends

Supplementary Figure 1. The *optix2/3-GAL4* line (*"optix>"*) drives expression in undifferentiated cells anterior to the morphogenetic furrow (MF). *optix2/3-GAL4; UAS-HA:tsh ("optix>HA:tsh")* L3 eye disc, stained for actin (Rhodamine-phalloidin, to outline tissue shape, red), HA, which tags *tsh* (green) and the photoreceptor marker Elav (blue). Anterior is to the left. Most of the HA:tsh driven by *optix2/3-GAL4* is detected anterior to the MF (line in A).

Supplementary Figure 2. (A) Principal Component analysis of the RNA-seq data from the five samples used. The analysis highlights the similarity of optix>tsh ("TSH") to the control, optix>GFP ("WT"), and between the two optix>hth+tsh biological replicates. optix>hth stands out as a different set. (B) MA-plot representing the log2 fold gene expression change of optix>hth+tsh over optix>GFP ("control") (y-axis), versus the abundance (x-axis). Red dots are differentially expressed genes. (C,D) Selected GO terms associated to genes that are down-regulated (C) or up-regulated (D) in optix>hth+tsh compared to the control optix>GFP.

Supplementary Figure 3. Co-expression of hth+tsh regulates the expression of cell cycle-related genes. GFP-marked clones overexpressing *hth+tsh* were induced in the eye imaginal disc at 48-72 hours after egg laying. Discs are stained with (A,A') anti-CycA, (B,B') anti-Dacapo and (C-C') anti-CycE (red). *hth+tsh*-expressing clones activate CycA and CycE expression and repress the expression of Dacapo.

Supplementary Figure 4. (A) Principal Component analysis of the FAIRE-seq data from the five samples used. (B) MA-plot representing the log2 fold gene expression change of optix>hth+tsh over control (y-axis), versus the abundance (x-axis). Red dots are differentially open peaks. (C) Association of FAIRE peaks (with or without Hth binding) to gene expression. (D1) Heatmap showing RNA-seq results for genes that show significantly closed FAIRE peaks and (D2, D3) genomic view of two examples. (E1) Heatmap showing RNA-seq results for genes that show significantly open FAIRE peaks and (E2 – E6) genomic view of five examples.

Supplementary Figure 5. i-cis-Target predicted transcription factor binding site motifs located within FAIRE peaks with increased accessibility in optix>hth+tsh discs.

Supplementary Figure 6. Adult phenotypes produce by the overexpression or RNAi-mediated attenuation of *Hr46/DHR3* and *ftz-f1.* Lateral and dorsal views of adult heads expressing different UAS lines using the optix2.3-GAL4 line: *DHR3RNAi* #412(A,A'), *DHR3RNAi* #413 (B,B'), *Hr46RNAi* #12044 (C,C'), *Hr46RNAi* #20157 (D,D'), *Hr46RNAi* #106837 (E,E'), *S-DHR3*(II) (F,F'), *S-DHR3*(III) (G,G'), *DHR3 RB* (H,H'), *ftz-f1RNAi* #33625 (I,I'), *ftz-f1RNAi* #104463 (J,J'), *ftz-f1RNAi* #2959 (K,K'), α *ftz-f1* (L,L'), β *ftz-f1* (M,M'), EcRB1 (N,N').

Supplementary Figure 7. Functional interaction of hth+tsh with *Hr46* and *ftz-f1.* Adult (upper panels) and eye disc (lower panels) phenotypes of *optix>hth+tsh+X,* with X being the UAS constructs indicated. Representative cases are shown.

Supplementary Figure 8. The dominant negative form of EcRB1 partially rescues the hth+tsh-phenotype. Late third instar eye discs from (A) *optix>GFP*, (B) *optix>GFP+EcRB1DN*, (C) *optix>hth+tsh+lacZ* or (D) *optix>hth+tsh+EcRB1DN* expressing flies. GFP in (A,B) comes from the UAS-GFP line and in (C,D) from the UAS-131-GFPhth line. The dashed lines outline the discs. (E) Statistical analysis of the GFP-positive area in the different genotypes.

Supplementary Figure 9. Temporal expression of Hr46. Hr46 expression during L3 development in the eye-antennal imaginal disc. Control disc at different developmental stages (early L3 (A), mid L3 (B) and late L3 (C)) stained with anti-Hr46. In (A) and (B) the dashed line outlines the disc.

Supplementary Figure 10. Visual representation of MEIS1 and TSHZ2 mRNA expression in (A) all 351 samples of the EXPO breast cancer dataset and (B) all 355 samples of the Sieber-Smith colon cancer dataset as analyzed using the R2 website. The tumors are ranked horizontally from left to right according to their MEIS1 expression as determined by Affymetrix array analyses (2log values). MEIS1 and TSHZ2 expression values for each tumor are visualized with black circles and red rectangles, respectively. Dark and light colors denote samples with significant ("present call"), and absent expression, respectively. mRNA expression correlations were calculated with a 2log Pearson test. Full details are in Supplementary Table 4. Visual representation of MEIS1 (C) and TSHZ2 (D) mRNA expression in the Finak breast cancer dataset, and MEIS1 (E) and TSHZ2 (F) DNA copy number levels in the TCGA-2 colon cancer dataset, as analyzed using the Oncomine website. Values are

represented as 2log-median centered. *P* values are calculated with a Student's t-test. Full details are in Supplementary Table 5.

Table legends

Supplementary Table 1. Upregulated and downregulated differentially expressed genes in optix>hth+tsh discs by RNA-seq.

Supplementary Table 2*.* FAIRE-seq analysis showing peak location and potential associated genes and Janelia lines overlapping peak regions.

Supplementary Table 3. Complete list of RNAi, UAS and dominant negative lines used for testing genetic interactions with hth+tsh.

Supplementary Table 4*.* MEIS1 and TSHZ1-3 mRNA expression and correlation in human cancer datasets.

(A) MEIS1 and TSHZ1-3 mRNA expression in human cancer datasets. Average MAS5.0-normalized data for mRNA expression, with their S.E.M., for the 116 different cancer datasets representing all major cancer types present in the R2 suite, 91 datasets for solid, and 25 for hematopoietic tumors. Only significant expression ("present call" for the probeset in that sample) is counted. Between brackets is the percentage of samples with such a present call. *MEIS1* and *TSHZ1* are almost invariantly well-expressed in solid tumor types, and also *TSHZ2-3* are significantly expressed in the majority of tumor samples. In hematopoietic tumors, *MEIS1* and *TSHZ1-3* are more rarely expressed, and their expression levels are lower, especially *TSHZ2-3*. For comparison: MAS5.0-normalized *GAPDH* and *ACTB* household gene mRNA expression in these datasets ranges between 5,000 and 10,000. (B) MEIS1 and TSHZ1-3 mRNA expression correlations in human cancer datasets. Cancer datasets in R2 with a significant correlation between *MEIS1* and *TSHZ1-3* mRNA expression levels. The number of sets with significant positive and negative expression correlations are listed in the top and bottom rows, respectively. In solid tumor types, MEIS1 is predominantly positively correlated with TSHZ1-3, most notably with TSHZ2-3, in hematopoietic tumors these correlations are also present, but much more infrequent. Complete data are in Supplementary Table 5. Correlations were calculated using a 2log Pearson test, as described in the Materials and Methods. (C) MEIS1 and TSHZ1-3 mRNA over-expression and DNA copy gain in human cancer types. Tumor

types are indicated in the first column. Examples of tumor subtypes with multiple datasets and consistent *MEIS1* and/or *TSHZ1-3* mRNA over-expression and/or DNA copy number gain in the Oncomine website are listed. The number of sets is indicated in the second column, the specific tumor subtypes in the third column. The last four columns list the number of datasets per tumor subtype that show significant mRNA over-expression and/or DNA gain. When in bold type and on a green background, *MEIS1* is also significantly positively correlated with *TSHZ1, -2,* or *-3* as analyzed in R2 (Supplementary Table 4). Various * enfolds 4 anaplastic large cell, 2 Burkitt's, 2 follicular, 3 Hodgkin's, 5 large B-cell, and 2 T-cell lymphoma datasets. Complete data are in Supplementary Table 6.

Supplementary Table 5. Complete MEIS1-TSHZ mRNA co-expression data from the R2 and used for Supplementary Table 3 and Supplementary Figure 10. Tumor type and dataset are listed in the first column, dataset size in the second column. Columns 3-9 show the R values of significant 2logPearson correlation tests for *MEIS1* mRNA expression correlation with the other genes listed. Results are from the probe-set with the highest correlation *R* value per dataset. Results are only listed if the best probe-set, or at least half of the other probe-sets showed significant correlations. Numbers on green and orange fields signify significant positive and negative correlations, respectively. Grey fields indicate that no probe-set was present. Column 10 lists the GEO GSE number, through which full set data can be obtained at the NCBI GEO website. (*)Available at http://cancergenome.nih.gov. (**)Available at http://www.ebi.ac.uk/arrayexpress. (***)Combination of GSE datasets 16910/18290/19348/21668. Results in bold red type are used as the examples in Supplementary Figure 10 (A, B).

Supplementary Table 6. Complete *MEIS1* and *TSHZ1-3* mRNA over-expression and DNA copy number gain data from Oncomine used for Supplementary Table 3C and Supplementary Figure 10. Shown are all tumor (sub)types with significant results. Tumor (sub)types with consistent MEIS1-TSHZ mRNA over-expression and/or DNA copy number gains in multiple datasets are on a green background. Tumor type and subtype are listed in the first two columns. Columns 3-6 show *P* value of mRNA over-expression (for mRNA datasets) or DNA copy number gain (for DNA datasets) in tumor over (matched) normal tissue, respectively. *P* values are as determined by a 2log-median centered t-test using the Oncomine default settings. Columns 7-9 show dataset name, type, and size, respectively. Columns 10 and 11 show amount of tumor, and normal tissue samples used in the t-test, respectively. Green fields indicate

consistent results for different datasets with the same tumor (sub)type. Results in bold red type are used as the examples in Supplementary Figure 10 (C-F).

Supplementary Table 7*.* Overview of MEIS1 co-expression with TSHZ, YAP1, RORA, and NR5A2 genes from R2 data. The first two columns list tumor type and amount of datasets. Columns 3-8 show whether at least half of the datasets showed consistent significant positive (POS; green field) or negative (NEG; orange field) mRNA expression correlations with MEIS1. Statistics and other details are as in Supplementary Table 5.

A

116 cancer sets				
	**MEIS1**	**TSHZ1**	**TSHZ2**	**TSHZ3**
**Solid tumors (n=91)**	**313 ± 35 (96%)**	**268 ± 17 (97%)**	**114 ± 7 (54%)**	**112 ± 6 (85%)**
**Hematopoietic tumors (n=25)**	**294 ± 74 (52%)**	**168 ± 14 (74%)**	**75 ± 11 (8%)**	**76 ± 6 (25%)**

B

91 solid cancer sets			
	**MEIS1-TSHZ1**	**MEIS1-TSHZ2**	**MEIS1-TSHZ3**
**Significant positive**	**46**	**56**	**60**
**Significant negative**	**6**	**2**	**7**

25 hematopoietic cancer sets			
	**MEIS1-TSHZ1**	**MEIS1-TSHZ2**	**MEIS1-TSHZ3**
**Significant positive**	**3**	**8**	**10**
**Significant negative**	**3**	**1**	**4**

C

Solid tumor						
**Tumor type**	**Sets**	**Subtype**	**MEIS1**	**TSHZ1**	**TSHZ2**	**TSHZ3**
Bladder	4	Bladder urothelial carcinoma	**3**		**3**	**4**
Brain	16	Astrocytoma, glioblastoma, oligodendroglioma	**10**	**7**	7	**4**
Breast	8	Breast carcinoma, invasive	**5**	1	**4**	**3**
Cervix	3	Cervical squamous cell carcinoma	**3**		3	1
Colon	18	Colorectal adenocarcinoma	**9**		**13**	**4**
Endometrium	2	Endometrial adenocarcinoma	**2**		**2**	
Esophagus	2	Esophageal adenocarcinoma				2
Germ cell	2	Teratoma, testicular	**1**	**2**	**1**	**1**
Head & Neck	3	Head & neck squamous cell carcinoma	**2**		**2**	1
Kidney	5	Renal cell carcinoma	**1**		**5**	**1**
Liver	6	Hepatocellular carcinoma	**4**	**1**	3	**3**
Lung	13	Lung adenocarcinoma, squamous cell carcinoma	**8**		**12**	**6**
Ovary	7	Ovarian adenocarcinoma, carcinoma	**7**		**1**	**1**
Pancreas	5	Pancreatic adenocarcinoma	**1**	1	**4**	4
Prostate	2	Prostate adenocarcinoma	**2**		2	**2**
Thyroid	1	Thyroid gland papillary carcinoma	**1**		**1**	1

Hematopoietic tumor						
**Tumor type**	**Sets**	**Subtype**	**MEIS1**	**TSHZ1**	**TSHZ2**	**TSHZ3**
Leukemia	8	Acute lymphoblastic leukemia	**6**	6	1	
Leukemia	7	Acute myeloid leukemia	7	2		1
Lymphoma	18	Various *	**17**	5	5	**13**
Myeloma	2	Multiple myeloma, smoldering myeloma		1		**1**
